# Profound Impact of Decline in N-Acetylgalactosamine-4-Sulfatase (Arylsulfatase B) on Molecular Pathophysiology and Human Diseases

**DOI:** 10.3390/ijms232113146

**Published:** 2022-10-29

**Authors:** Joanne K. Tobacman, Sumit Bhattacharyya

**Affiliations:** Jesse Brown VA Medical Center, University of Illinois at Chicago, Chicago, IL 60612, USA

**Keywords:** N-acetylgalactosamine-4-sulfatase, Arylsulfatase B, chondroitin 4-sulfate, dermatan sulfate, sulfated glycosaminoglycans, mucopolysaccharidosis, cystic fibrosis, malignancy, proteoglycans, Warburg effect

## Abstract

The enzyme N-acetylgalactosamine-4-sulfatase (Arylsulfatase B; ARSB) was originally identified as a lysosomal enzyme which was deficient in Mucopolysaccharidosis VI (MPS VI; Maroteaux-Lamy Syndrome). The newly directed attention to the impact of ARSB in human pathobiology indicates a broader, more pervasive effect, encompassing roles as a tumor suppressor, transcriptional mediator, redox switch, and regulator of intracellular and extracellular-cell signaling. By controlling the degradation of chondroitin 4-sulfate and dermatan sulfate by removal or failure to remove the 4-sulfate residue at the non-reducing end of the sulfated glycosaminoglycan chain, ARSB modifies the binding or release of critical molecules into the cell milieu. These molecules, such as galectin-3 and SHP-2, in turn, influence crucial cellular processes and events which determine cell fate. Identification of ARSB at the cell membrane and in the nucleus expands perception of the potential impact of decline in ARSB activity. The regulation of availability of sulfate from chondroitin 4-sulfate and dermatan sulfate may also affect sulfate assimilation and production of vital molecules, including glutathione and cysteine. Increased attention to ARSB in mammalian cells may help to integrate and deepen our understanding of diverse biological phenomenon and to approach human diseases with new insights.

## 1. Introduction

N-acetylgalactosamine-4-sulfatase or Arylsulfatase B (ARSB; EC 3.1.6.12) is the sulfohydrolase which specifically removes the 4-sulfate group from N-acetylgalactosamine 4-sulfate residues, such as those at the non-reducing end of the sulfated glycosaminoglycan (GAG) chains of chondroitin 4-sulfate (C4S) and dermatan sulfate (DS). This enzyme is required for the degradation of these GAGs [1,2,3,4,5]. The purpose of this review is to provide a summary of the known associations of decline in ARSB with human diseases and to consider the underlying biochemical mechanisms which lead to the pathobiology. Since the effect of ARSB is limited to modifications of N-acetylgalactosamine 4-sulfate residues, the manifestations of decline in ARSB evolve from failure to remove the 4-sulfate group and from the accumulation of the undegraded substrates. This specificity permits focus on distinct pathways and interactions which have the potential to lead to new insights into how a broad range of biological effects may originate.

Inherited mutations of ARSB are the cause of Mucopolysaccharidosis (MPS) VI (Maroteaux-Lamy-Syndrome; phenotype MIM number is 253200) [6,7,8,9,10,11], which is characterized by the accumulation of dermatan sulfate and chondroitin 4-sulfate (C4S) throughout the body. Manifestations of include short stature, skeletal deformities, hepatomegaly, respiratory failure, corneal opacities, dental anomalies, reduced life expectancy, and normal intellect [10]. Pathophysiology attributable to decline in ARSB extends beyond MPS VI and is associated with other human diseases, including multiple sulfatase deficiency, cystic fibrosis, malignancies and neurologic, cardiac, and respiratory disorders, as presented in Section 14. These effects are generally attributable to the accumulation of excessive, undegradable sulfated GAGs and the resulting impact on cell signaling, transcription, differentiation, and metabolism, as detailed in Section 5, Section 6, Section 7, Section 8, Section 9, Section 10, Section 11, Section 12 and Section 13.

Human ARSB transcript variant 1 (NM_000046; NP_000037; OMIM 611542) is composed of 533 amino acids, and transcript variant 2 (NM_198709; NP_942002) is 413 amino acids and is truncated at the 3′ end. Signal peptide is amino acids 1–36, and cysteine 91 is the crucial residue for the post-translational modification and activation of ARSB [12]. N-acetylgalactosamine-4-sulfate residues, specifically those of chondroitin 4-sulfate (C4S) and dermatan sulfate (DS), are the endogenous substrates of ARSB. C4S is composed of repeating disaccharides of D-glucuronate linked β-1,3 to D-N-acetylgalactosamine-4-sulfate. Dermatan sulfate (DS) is composed of repeating disaccharides of D-iduronate linked β-1,3 to D-N-aceylgalactosamine-4-sulfate. The disaccharides are joined by β-1,4 linkages. These sulfated glycosaminoglycans are abundant in mammalian tissues, present at concentrations of up to hundreds of micrograms per g wet weight of tissue [13,14].

The ARSB crystal structure was reported in 1997, revealing the active site cysteine C91 as the 3-oxoalanine [also designated as C-formylglycine (FGly)] derivative [15]. The sulfate in the crystallized structure is bound to calcium, and resemblance to alkaline phosphatase was evident at the active site and by the resemblance of the calcium binding site to the zinc binding site in alkaline phosphatase [15]. Earlier work had well-characterized structural features of ARSB [16,17,18]. The structure is complex, with multiple alpha helices, beta sheets, glycosylation sites, and disulfide bonds; molecular weight is 59,687 Da. Production of antibodies for measurements of ARSB facilitated identification of ARSB in tissues and urine [17]. Mutations of the C91 residue of ARSB showed loss of activity, al-though with retained polypeptide content [18]. The ARSB gene was located at 5q14.1, and a 2.2 kilobase cDNA clone for the human ARSB transcript was isolated [19,20]. Genomic coordinates (GRCh38) from NCBI are: 5:78,777,209–78,985,958. Promoter activity is in a 398 bp 5′-flanking region.

The activation of ARSB, similar to other sulfatase enzymes, requires post-translational modification by the formylglycine modifying enzyme (FGE) [21,22], the product of the *SUMF* (sulfatase-modifying factor) gene [23,24]. The formylglycine modification of the critical cysteine residue (cysteine 91) of ARSB by the FGE requires molecular oxygen [25]. Crystallization of the FGE and ARSB have enabled clarification of the specific requirements for their interaction [22,25], including identification of sequence determinants required for conversion of cysteine91 to formylglycine [26].

When removal by ARSB of the 4-sulfate group at the non-reducing end of C4S is impaired, altered binding of critical, signaling molecules to chondroitin 4-sulfate (C4S) results. (Similar effects may occur with dermatan sulfate, but our studies presented in this report have focused on C4S, due to availability of materials.) Important, specific changes include the reduced binding of the galactoside-binding protein galectin-3 (LGALS3) [27] and the increased binding of SHP2 (PTPN11), the ubiquitous, non-receptor tyrosine phosphatase, to C4S when ARSB activity is lower and chondroitin 4-sulfation is increased [14,28]. Other reported effects of changes in ARSB on binding of vital molecules with C4S include increased binding of Interleukin (IL)-8 [29] when ARSB is reduced; increased binding of bone morphogenetic protein (BMP)-4 [30] when ARSB is reduced; and reduced binding of high molecular weight kininogen (HMWK) [31] to the less highly sulfated C4S present when ARSB activity is overexpressed. The increased chondroitin 4-sulfation affects the phosphorylation of vital signaling molecules and the regulation of transcriptional events, as detailed in this review [14,27,28,29,30,31,32,33,34,35,36,37,38]. These events profoundly impact intra- and extra-cellular signaling, influencing cell proliferation, differentiation, signaling, transcription, and transformation, as indicated in Section 5, Section 6, Section 7, Section 8, Section 9, Section 10, Section 11, Section 12 and Section 13. The impact of ARSB and the resulting changes in chondroitin 4-sulfation on human disease and the underlying molecular mechanisms, so far as currently elucidated, are discussed in detail in this review.

## 2. Extra-lysosomal Localization of ARSB

**Figure 1:**
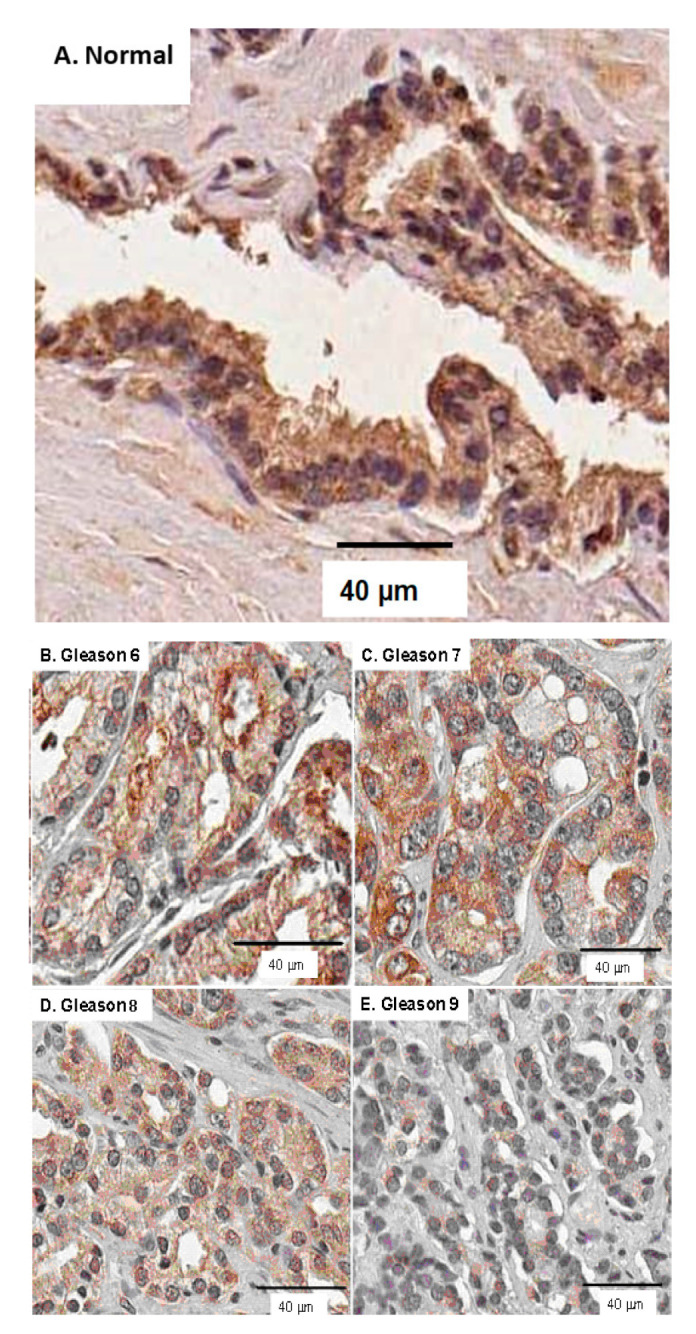
Representative images of normal and malignant prostate cancers, immunostained for ARSB. (**A**–**E**). Declines in intensity and in membrane immunostaining are evident, as Gleason score increases from 6 to 9 [42].

**Figure 2:**
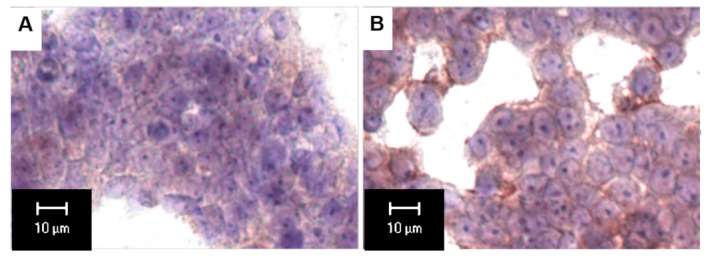
ARSB immunostaining of malignant and non-malignant human colonic epithelial cell lines. The malignant T84 cells (**A**) have markedly lower immunohistochemical intensity than the NCM460 cells (**B**). Cell membrane immunostaining is prominent in the non-malignant cells [34].

**Figure 3:**
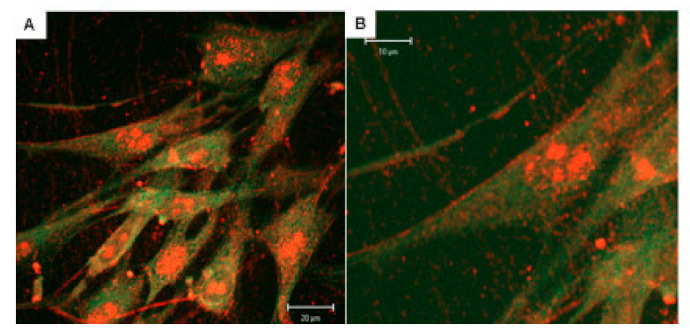
Fluorescent immunostaining of ARSB in human cerebrovascular cells. (**A**,**B**). Confocal microscopy demonstrates cell surface localization of ARSB in untreated cerebrovascular cells, as well as cytoplasmic and nuclear localization and presence of ARSB in cell projections. ARSB is stained red, and β-actin is stained green [44].

Although originally considered only a lysosomal enzyme, immunohistochemistry, immunofluorescence, and activity studies localized ARSB at the cell membrane of human bronchial epithelial cells [29,39], hepatocytes, sinusoidal endothelial cells, and Kupffer cells in the mammalian liver [40], apical membranes of normal and malignant human colonic and prostatic epithelial cells [34,41,42,43], and human cerebrovascular cells [44]. Representative images show staining of normal and malignant prostate tissues (Figure 1A–D), human colonic epithelial cells (Figure 2A,B), and human cerebrovascular cells (Figure 3A,B).

Other reported extra-lysosomal sites of ARSB include alpha-granules of platelets [45], mitochondria of rat kidney proximal convoluted tubule epithelial cells [46], surface membranes of cartilage [47], and rat brain membranes [48,49,50]. Arylsulfatase B is very widely distributed in most human tissues, with high expression reported in kidney, cervix, and heart [51]. Our initial studies demonstrated higher measured ARSB enzyme activity in cell membrane preparations from human bronchial epithelial cells than in the cytosol [39]. Presence of ARSB activity in the cell membrane suggests that ARSB may act in close proximity to chondroitin 4-sulfate or dermatan sulfate in the extracellular matrix or at the membrane. ARSB activity was also detected in nuclei and mitochondria [39,52]. The sorting mechanism whereby ARSB is directed to lysosomes, or to the cell membrane, or to other sites, such as mitochondria, is unknown.

ARSB immunostaining in a human colonic microarray showed differences in distribution, intensity, and pattern of ARSB staining among normal colon, adenomas, and adenocarcinomas [41]. Distinctive, intense luminal membrane staining was present in the normal epithelial cells with a prominent pattern of intense positivity at the luminal surface and reduced staining deeper in the crypts. Prominent immunostaining of ARSB was also detected in human lung tissue, both in the cytoplasm and along the cell periphery [29]. Confocal microscopy of cerebrovascular cells also demonstrated prominent membrane, cytosolic and nuclear ARSB staining, as shown in Figure 3A,B [44].

When Mitsunaga-Nakatsubo and co-workers examined hepatic localization of ARSB in the liver using light and electron microscopy, they found ARSB on the cell surface of sinusoidal endothelial cells, hepatocytes, and sinusoidal macrophages (Kupffer cells), as well as in the lysosome [40]. ARSB colocalized with proteoglycan, and they concluded that ARSB functioned in the cell surface of mammals.

## 3. Measurement of ARSB Activity

Assays for measurement of ARSB activity were initially developed using the synthetic substrate, p-nitrocatechol sulfate (NCS), and subsequently with 4-methylumbelliferyl sulfate (MUS) [53,54]. The fluorometric assay with 4-MUS requires smaller volumes of cells and is detailed in Table 1. Other assays have used the endogenous chondroitin 4-sulfate substrate or other exogenous sulfated substrates in commercial ELISAs [55,56]. Many commercial ARSB antibodies are now available, but the antibodies may or may not distinguish between functional vs. non-functional ARSB. In large-scale screening assays, ARSB has been detected in dried blood spots [57,58]. Disaccharide analysis provides precise information about the impact of ARSB activity on production of unsulfated vs. 4-sulfated disaccharides in cell or tissue samples [29,38,44].

Distinguishing between effects of ARSB and Arylsulfatase A (ARSA; cerebroside sulfatase) has been challenging for investigators. ARSB was distinguished from ARSA by differences in effectiveness of hydrolysis of tyrosine sulfates, since ARSB was only 5% as effective as ARSA [59]. Refinement of the assay for ARSB vs. ARSA required using specific inhibitors of ARSA, such as barium sulfate or silver nitrate [60,61,62].

## 4. Inhibition of ARSB Activity by Ions, Metals, Hormones, Chemicals, and Hypoxia

In the literature, several inhibitors of ARSB activity are reported, including chloride, phosphate, sulfate, sulfite, and ascorbic acid [54,62,63,64,65,66,67,68,69,70]. Table 2 lists inhibitors of ARSB, as presented in this section.

### 4.1. Inhibition by Chloride, Phosphate, Sulfate, and Sulfite Ions

Chloride exposure was reported to inhibit ARSB activity in early reports [64,65,66]. Increase in exposure to exogenous chloride (NaCl) produced significant declines in the ARSB activity of normal rat kidney epithelial cells (NRK-E52, ATCC), from 158.0 ± 8.5 to 122.2 ± 4.3 nmol/mg protein/h with 75 mM chloride and to 79.2 ± 4.6 nmol/mg protein/h with 100 mM chloride) [70]. In contrast, exposure to varying concentrations of Na-acetate (from 0 to 100 mmol/L) had no effect on the ARSB activity. In addition, ARSB activity in the renal tissue of salt-sensitive rats exposed to a high salt (NaCl) diet was significantly less than in rats following a low salt-diet [31].

Phosphate was reported to inhibit activity of ARSB [63,67,68], and addition of phosphate-buffered saline (PBS) reduced the measured ARSB activity in bronchial epithelial cells [39]. When PBS was substituted for Na-acetate, a PBS concentration of 0.125 M reduced ARSB activity by more than 95%. However, the impact of cellular phosphate on modulation of ARSB activity in vivo is unknown. The combination of pyrophosphate and 1 M NaCl was also reported to inhibit ARSB [68]. Sulfate and sulfite have also been reported to inhibit activity of ARSB [64,67,68].

### 4.2. Effects of Metals

The inhibitory effect of metallic ions on the activity of ARSB was considered and used to distinguish activity of ARSB vs. ARSA [60,61,62,64]. Lead had no inhibitory effect on human ARSA and enhanced ARSB activity. Barium ion inhibited ARSA, with no apparent effect on ARSB. Silver ion negligibly affected ARSB in dialyzed human serum, but completely inhibited the activity of dialyzed human urinary ARSA. Vanadium has also been identified as an inhibitor of ARSB and was represented in the structural model of ARSB [15].

### 4.3. Inhibition by Hormonal Exposures

The impact of hormonal exposures on ARSB activity was examined in studies of ventral rat prostate development [69,71], human prostate epithelial cells [37], and human mammary cell lines [70,72].

#### 4.3.1. Estrogen Exposures

Baseline ARSB activity increased significantly between 5 and 30 days in ventral rat prostate tissue [71]. Following estrogen exposure (estradiol benzoate 25 μg in 25 μL sesame oil subcutaneously on days 1, 3, and 5), ARSB activity declined, and the baseline increase on day 30 was inhibited. (In contrast, estrogen treatment did not block the increase in GALNS activity between days 5 and 30.)

Effects of estrogen were also detected in studies of cultured human mammary cells [72]. In MCF-7, T47D, and MCF10A cell lines (from ATCC), and in primary human myoepithelial cells, treatment with estrone (100 pg/mL), estradiol (200 pg/mL), estrone 3-sulfate (1.5 ng/mL) or estradiol sulfate (3.0 ng/mL) for 2–6 days significantly reduced ARSB activity. No changes in ARSB were detected in normal, primary human epithelial cells or in the HCC1037 mammary cell line.

#### 4.3.2. Androgen Exposure

In cultured normal prostate epithelial cells (CRL-2850, ATCC), treatment with dihydrotestosterone (10 nM × 24 h) reduced ARSB gene expression due to increased ARSB promoter methylation [37].

### 4.4. Chemical Inhibitors of ARSB: Ascorbate, Carrageenan, Chloroquine, and Ethanol

Ascorbate was reported to inhibit activity of ARSB, with progressive decline in activity of cultured chondrocytes with increasing levels of ascorbic acid [69].

Exposure of cultured human colonic epithelial cells (primary and NCM460 cells, INCELL, San Antonio, TX, USA) to the common food additive carrageenan, which is composed of sulfated or unsulfated galactose residues in alternating beta-1,4 and alpha-1,3 bonds, led to declines in ARSB activity [73,74]. This effect may be attributable to carrageenan’s mimicry of the endogenous 4-sulfated glycosaminoglycans, dermatan sulfate and chondroitin 4-sulfate [73].

The treatment of human cerebrovascular cells, placental cells, and bronchial epithelial cystic fibrosis cells, corrected or uncorrected for CFTR, by chloroquine (50 nM × 24 h), significantly reduced the ARSB activity, protein, and mRNA [44]. This finding is relevant to multiple studies which demonstrate that more highly sulfated chondroitin 4-sulfate reduces infectivity by malarial parasites [76,77,78,79].

Ethanol was shown to inhibit ARSB activity in primary astrocytes prepared from rat neocortex and from rat hippocampus in a dose-dependent manner [68,75]. Maximum effect on activity occurred at 75 mM concentration, although ARSB mRNA expression was unaffected.

### 4.5. Inhibition by Hypoxia

The activation of ARSB requires the formylglycine-generating enzyme (FGE) and molecular oxygen [21,22]. In human bronchial epithelial cells (BEC) and colonic epithelial cells following exposure to 10% oxygen, ARSB activity declined significantly by 0.25 h and remained low for 24 h, in comparison to control cells under normoxic conditions [35]. Return to normoxia for 4 h after 4 h of 10% O_2_ restored the baseline ARSB activity. Hypoxia produced no decline in sulfatase modifying factor (*SUMF*)*-1*, the gene for the FGE. Silencing *SUMF-1* by siRNA significantly reduced the ARSB activity, and maximum reduction in ARSB activity was achieved by the combination of *SUMF-1* silencing and hypoxia.

Acquired deficiency of ARSB due to exposures, such as to ethanol, carrageenan, chloroquine, hormones, or other factors, may lead to accumulation of C4S and DS. Effects of decline in ARSB on the monosaccharide N-acetylgalactosamine 4-sulfate have not yet been associated with any specific pathophysiology, but may contribute to cell-cell recognition and signaling. Decline in ARSB may reduce the availability of sulfate, with potential impact on cell metabolism, ion exchange, and cell signaling.

## 5. Arylsulfatase B and Chondroitin 4-Sulfation Regulate Signaling Mechanisms

**Figure 4:**
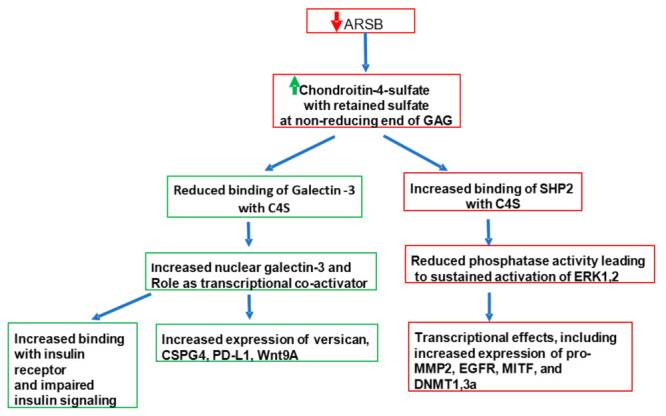
Mechanisms leading to impaired cell signaling and transcriptional events. Decline in ARSB leads to accumulation of chondroitin 4-sulfate and dermatan sulfate. Decline in ARSB may be due to congenital mutation, hypoxia, increased exposure to chloride or phosphate, estrogen, ethanol, carrageenan, or other exposures, as indicated in Table 2 and Section 4. The signaling pathways were explored with chondroitin 4-sulfate (C4S), since a C4S antibody which detected C4S chains was available. Two major pathways are affected by increased C4S, as shown. Galectin-3 binds less to more highly sulfated C4S and becomes available for other interactions, including increased binding with the insulin Receptor, leading to inhibition of insulin signaling [80,81,82] and increased binding with transcription factors, enabling increased DNA binding and transcriptional effects [27,32,33,34,35]. In contrast, SHP2 binds more with C4S when ARSB is silenced, leading to reduced phosphatase activity [14,28,33,36,37,38] and sustained phosphorylation of critical signaling molecules, including phospho-ERK1,2 [33,36,83], phospho-JNK [28], and phospho-p38 MAPK [14,38]. Due to the complexity of the phospho-proteome, the manifestations of sustained phosphorylations impact multiple signaling events. These include increased c-Myc activation of DNMTs and increased methylation of DKK3, which when active acts as an inhibitor of Wnt signaling [36,37,38].

The influence of Arylsulfatase B (ARSB) on cell signaling has been explored through changes in chondroitin 4-sulfate when ARSB is silenced by siRNA in normal and malignant human cell lines and tissues and in tissues of the ARSB-null mouse. Two major effects are due to altered binding with more highly sulfated chondroitin 4-sulfate present when ARSB is reduced; galectin-3 binding is reduced [27,32,33,34,35] and SHP-2 binding is enhanced [14,28,33,36,37,38]. Figure 4 schematically presents these pathways.

### 5.1. Activation of Galectin-3 (LGALS3)

By frontal affinity chromatography, the β-galactoside binding proteins galectins 3, 7, and 9 were shown to bind preferentially to desulphated galactosaminoglycans, rather than more highly sulfated C4S or DS [84]. When ARSB was silenced and chondroitin 4-sulfation thereby increased, galectin-3 binding with C4S declined and nuclear galectin-3 increased in human prostate epithelial and stromal cells, human colonic cell lines, and control and ARSB-null mouse colonic epithelium, and human bronchial epithelial cells [27,32]. In colonic epithelial cells, galectin-3 translocated to the nucleus and facilitated the binding of transcription factor Sp1 with the Wnt-9A promoter, leading to increased expression of Wnt-9A [32]. In experiments in prostate stromal and epithelial cells, when ARSB was silenced, binding of galectin-3 with C4S declined, nuclear galectin-3 increased and facilitated the binding of transcription factor AP-1 to the versican promoter [27]. Increased expression of CSPG4 (chondroitin sulfate proteoglycan 4) [33] and of PD-L1 [85] in human melanoma cells are also dependent on galectin-3. Expression of HIF-1α in human bronchial epithelial cells and in the colonic epithelial cell line NCM460 increased when ARSB was silenced and declined when ARSB was overexpressed [35]. When ARSB was silenced by siRNA or cells were exposed to hypoxic conditions (10% O_2_ × 4 h), the galectin-3 co-immunoprecipitated with C4S antibody (4D1, SCBT; not an antibody to C4S stubs) declined and nuclear galectin-3 increased [35].

### 5.2. Inhibition of SHP2 Activity

In addition to impact on galectin-3 and associated transcriptional effects, when ARSB was silenced and chondroitin 4-sulfation thereby increased, binding to C4S of the non-receptor, ubiquitous tyrosine phosphatase SHP2 (PTPN11) increased and SHP2 activity declined in prostate cell lines (CRL-2854, CRL-2850, CRL-2887, ATCC) [14,28,33,36,37,38]. This effect was reduced in the presence of chondroitin 6-sulfate [28]. Notably, when the SHP2 small molecule inhibitor PHPS1 (phenylhydrazonopyrazolone sulfonate) was developed, the SHP2/PHPS1-binding model demonstrated that the phenyl sulfonate group of PHPS1 acted as a phosphotyrosine mimetic [86] and the sulfonate group penetrated into the substrate-binding pocket of SHP2. The sulfate group of GalNAc 4-sulfate at the non-reducing end of C4S may also act as a phosphotyrosine mimetic and penetrate into the substrate binding pocket of SHP2 in prostate epithelial and stem cells [28,36]. The amino acids at the periphery of the cleft where the PHPS1 sulfate binds with SHP2 are Lys-280, Asn-281, Arg-362, and His-426. These residues, which are distinct in SHP2, may act to stabilize the anionic sulfate of C4S and maintain SHP2 in a bound, inactive conformation. The 4-sulfate group at the non-reducing end of C4S may be a unique configuration; it has previously been shown to be critically important in binding of malarial parasites in the vasculature [76,77,78,79]. Thus, the retained 4-sulfate group at the non-reducing end of C4S when ARSB is reduced may provide a niche for SHP2 interaction, leading to SHP2 inactivation, and sustained activation of ERK1/2 [33,36], JNK [28], and p38-MAPK [14,38] and thereby impact on critical intracellular signaling pathways.

## 6. Impact on Transcriptional Events

**Figure 5:**
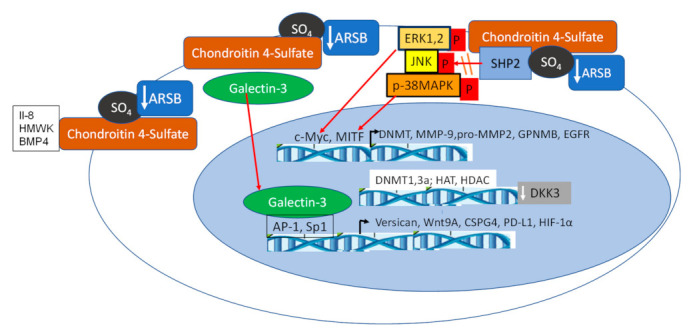
Overall schematic of transcriptional effects following decline in ARSB. Decline in ARSB leads to increase in chondroitin 4-sulfation, due to failure to remove the sulfate group at the non-reducing end and inhibition of degradation, as occurs in the inherited disorder MPS VI. Increased binding to C4S occurs for several vital molecules, including IL-8 [29] and BMP-4 [30]. Sequestration of IL-8 in the cell membrane can lead to increased neutrophil chemotaxis and contribute to impaired mucociliary clearance. BMP4 retention with C4S can alter Wnt-BMP interactions and reduce the Smad-mediated expression of CHST11 [30], thereby impairing production of new C4S. Galectin-3 binds less to more highly sulfated C4S, leading to availability for binding with the insulin receptor, and thereby contributing to insulin resistance [80,81,82]. In addition, galectin-3 acts as a co-transcriptional activator, combining with AP-1 and Sp1 for enhanced transcription of versican [37], CSGP4 [33], HIF-1α [35], Wnt9A [32], and PD-L1 [85]. In contrast, SHP2 binds more with C4S when ARSB is inhibited, leading to decline in phosphatase activity and sustained phosphorylation of important mediators, including phospho-ERK [33,36,83], phospho-JNK [28], and phospho-P38 MAPK [14,38]. Through a network of signaling events, nuclear c-Myc [36] and MITF [14] and other transcription factors act to increase expression of pro-MMP2 [33], MMP9 [33,83], GPNMB [14], EGFR [28], and DNMT1 and 3a [36]. Hence, a broad range of vital cellular processes are regulated due to changes in ARSB activity and chondroitin-4 sulfation.

Decline in ARSB modifies transcription events by several mechanisms, including effects related to changes in galectin-3 and SHP2 activity, as described above. Specific effects include: (1) enhanced methylation leading to activation of Wnt signaling [36]; (2) modification of histone acetylation due to effects on HDAC and HAT activity (unpublished data); (3) altered DNA binding of transcription factors, including AP-1, Sp1, GATA-3, MITF [12,14,27,28,38]; and (4) increased expression of transcription factors, including c-Myc, Gli, cyclin D1, and TCF/LEF [36,37,84]. Figure 5 presents these transcriptional effects. 

### 6.1. Increased DNA Methylation and Disinhibition of Wnt/β-Catenin Signaling

The mechanism by which decline in ARSB activates Wnt/β-catenin signaling in cultured prostate epithelial cells is due to increased expression and activity of DNA methyltransferases (DNMT) [36,37] leading to reduced expression of DKK3 (Dickkopf Wnt pathway signaling inhibitor 3) and the disinhibition of Wnt signaling. Following ARSB silencing by siRNA in cultured human prostate epithelial cells and in malignant prostate tissue, DNMT activity and expression of DNMT1 and DNMT3a increased, with no change in DNMT3b expression. These increases followed decline in SHP2 activity, increased ERK1/2 phosphorylation, and increased DNA binding of c-Myc/Max. Increased methylation of the DKK3 promoter occurred, and reduced expression of DKK3 led to the disinhibition of Wnt signaling following exposure to Wnt3a in prostate epithelial cells. Inhibition of methylation by treatment with 5-azacytidine reversed the decline in DKK3 expression and activated Wnt signaling, as shown by increases in nuclear β-catenin and TCF/LEF DNA binding. Interestingly, effects of ARSB silencing were similar to those of GALNS overexpression, leading to decline in chondroitin 6-sulfation. This is consistent with inhibition by chondroitin 6-sulfate of the binding of SHP2 with more sulfated C4S following decline in ARSB.

### 6.2. Effects on Histone Acetylation/Deacetylation Activity

Recent work (unpublished) in melanoma cell lines reveals inverse effects of ARSB silencing and treatment by rhARSB on the activity of histone acetyltransferases (HAT) and on histone deacetylases (HDAC). Clarification of the impact of these findings on transcription is ongoing.

### 6.3. Increased Expression of Transcription Factors

In cultured human prostate stem cells, ARSB silencing, and overexpression significantly modified the expression of several transcription factors in a transcription factor array [87]. Inverse changes between ARSB silencing vs. ARSB overexpression occurred for TCF/LEF, c-Myc, and Gli. In human bronchial and colonic epithelial cells, ARSB silencing, and overexpression also had inverse effects on expression of HIF1α [35]. When human bronchial epithelial and intestinal epithelial cells were exposed to ambient oxygen concentration of 10% or silencing of ARSB, expression of HIF-1α increased. Inversely, when ARSB was overexpressed in these cells, HIF-1α expression declined. The combination of hypoxia and ARSB silencing had similar effect as ARSB silencing alone. The transcription factors GATA-3, cyclin D1, and TCF/LEF were increased in prostate, and MITF was increased following ARSB silencing in HepG2 cells and in hepatic tissue from ARSB-null mice [14,36,37].

### 6.4. Increased DNA Binding of Transcription Factors

Experiments demonstrated increased DNA binding of several transcription factors (TF) following silencing of ARSB, including TCF/Lef [36,87], AP-1 (c-Jun/c-Fos) [27,28], Sp1 [32], MITF [14], c-Myc/Max [36], HIF-1α [35], and GATA-3 [38]. These TFs were associated with increased expression of cyclin D1, versican, Wnt9A, GPNMB, DNMT, and CHST15.

## 7. Impact on Motility and Invasiveness

Increased expression of pro-MMP2 and MMP-9 occurred in melanoma cell lines following ARSB silencing, and these increases were associated with increased invasiveness [33]. Treatment with rhARSB reduced the invasiveness of the cells, as detected in an invasiveness assay. In human bronchial epithelial cells, expression of MMP-9 was increased when ARSB was lower, as in uncorrected cystic fibrosis (CF) cells, or following ARSB silencing [83]. In human colonic epithelial cells (T84, NCM460 and normal colonocytes), decline in ARSB increased the expression of MMP-9, and expression was reduced by ARSB overexpression, perhaps attributable to a RhoA-mediated mechanism [34]. Cell migration in response to 10% FBS increased when ARSB was silenced, and, inversely, overexpression of ARSB reduced the cell migration. In melanoma cells, decline in ARSB increased expression of MMP-9 and pro-MMP2 due to inhibition of SHP2 and activation of phospho-ERK1/2. In bronchial epithelial cells, ERK inhibition blocked the increase in MMP-9 which followed interaction of GPNMB with β-1 integrin [83].

## 8. Impact of Decline in ARSB on Activation of Phospho-ERK1/2, Phospho-JNK and Phospho-p38 MAPK

In human cell lines and tissues and in tissues of the ARSB-null mouse, decline in ARSB is associated with increased chondroitin 4-sulfation leading to inhibition of SHP2 activity and sustained phosphorylation and activation of critical kinase signaling pathways involving phospho-ERK1/2 [33,36,83], phospho-JNK [28], and phospho-38 MAPK [14,38].

In human melanoma cells, expression of pro-MMP2 and MMP-9 was increased following ARSB silencing and was mediated by decline in SHP2 activity and increase in phospho-ERK1/2 [33]. In bronchial epithelial cells, MMP-9 expression increased when phospho-ERK1/2 increased following interaction between GPNMB (transmembrane glycoprotein NMB) and β-1 integrin [83]. In prostate epithelial cells, decline in SHP2 when ARSB was silenced led to increased phospho-ERK1/2 and enhanced c-Myc nuclear binding, DNMT activity and expression of DNMT1 and DNMT3a and reduced expression of DKK3, leading to disinhibition of Wnt signaling [36].

In cultured prostate epithelial and stem cells, decline in ARSB increased the expression of EGFR [28]. The pathway of this increase required increased phospho-JNK and nuclear c-fos binding to the EGFR promoter, following decline in SHP2 activity due to increased binding with C4S.

In HepG2 cells, silencing ARSB increased phospho-p38 MAPK and nuclear MITF, leading to increased GPNMB expression [14]. These effects followed inhibition of SHP2 activity by increased C4S, and effects were reversed by ARSB overexpression. Phospho-p38 MAPK increased following exposure to Wnt3A in prostate epithelial cells, and this increase was blocked by inhibition of Rac-1 GTPase [38]. In these cells, increased phospho-p38 MAPK led to nuclear translocation of GATA-3 and increased expression of CHST15.

## 9. Increased Expression of Proteins Vital to Inflammation, Inter-Cellular Signaling, and the Immune Response

Decline in ARSB and the associated increase in chondroitin 4-sulfation and impact on transcriptional events lead to increased expression of critical molecules, including proteins involved with inflammation, immunogenicity, and cell-cell signaling.

### 9.1. Increased Interleukin-6 (IL-6)

In CF patients and asthmatic patients with lower ARSB in their circulating leukocytes, serum IL-6 levels were markedly increased, consistent with the observed decline in leukocyte ARSB [88]. In human bronchial epithelial cells, correction of CFTR by a potentiator which normalized ARSB activity was associated with decline in IL-6 (Interleukin-6) secretion [89]. IL-6 expression was increased in bronchial epithelial cells in a hypoxic gene array when ARSB was silenced, or cells were exposed to 10% O_2_ for 4 h [35].

### 9.2. Increased GPNMB Expression

Experiments in HepG2 cells, melanoma cell lines, normal melanocytes, and hepatic and prostate tissue of ARSB-deficient mice revealed that decline in ARSB was associated with increase in expression of GPNMB (Glycoprotein Nonmetastatic Melanoma Protein B; Glycoprotein (Transmembrane) Nmb; osteoactivin) [14]. Membrane-associated GPNMB binds with extracellular β1 integrin and induced the phosphorylation of ERK1/2, leading to other transcriptional events, including increased expression of MMP-9 [83]. GPNMB has been shown to augment tumor growth and metastasis and to be overexpressed in cancers, enhancing tumor cell proliferation, migration, and invasion [90,91].

### 9.3. Increased Expression of Wnt9A (Wnt14)

In human colonic epithelial cells, when ARSB was silenced or cells were treated with carrageenan to reduce ARSB activity, the expression of Wnt9A increased [32]. Wnt9A has been associated with colorectal, pancreatic, and gastric carcinomas [92,93].

### 9.4. Increased Programmed Death-Ligand 1 (PD-L1)

In human melanoma cell lines and melanoma tissue, decline in ARSB increased PD-L1 (programmed death ligand-1) expression [85]. Silencing ARSB by siRNA also increased PD-L1 expression in human prostatic and hepatic cell lines. Treatment by rhARSB in melanoma cell lines reduced the PD-L1 expression (unpublished data). In other experiments, PD-L1 gene and protein expression declined in B16F10 melanomas in C57BL/6J mice following treatment with recombinant ARSB (unpublished data). These findings suggest that modification of ARSB may have profound effects on immune cell-epithelial cell interactions.

## 10. Regulation of Secretion of Critical Molecules due to More or Less Binding with Chondroitin 4-Sulfate

### 10.1. High Molecular Weight Kininogen Binding with Chondroitin 4-Sulfate Is Reduced by Increase in ARSB

When Dahl salt-sensitive (SS) rats were exposed to high and low salt diets, ARSB activity was significantly less in the renal tissue of the high salt-fed rats than in the renal tissue of the low salt-fed rats [70]. Correspondingly, chondroitin-4-sulfate and total sulfated glycosaminoglycan content were significantly greater in the rats on high salt. Disaccharide analysis confirmed marked increase in C4S disaccharides in the renal tissue of the high salt-fed rats. In contrast, unsulfated, hyaluronan-derived disaccharides were increased in the rats on the low salt diet. In the high salt-fed rats, with lower ARSB activity and higher chondroitin 4-sulfation, cell-bound, high-molecular weight kininogen was greater and urinary bradykinin was lower. Experiments demonstrated a reduction in cell-bound high molecular weight kininogen and increase in bradykinin secretion in normal rat kidney (NRK) epithelial cells when chondroitin-4-sulfate content was reduced following overexpression of ARSB [31].

### 10.2. Interleukin-8 Secretion Declined due to Increased Sequestration with C4S When ARSB Is Lowered, Enhancing Neutrophil Chemotaxis

In experiments with the cystic fibrosis cell line IB3-1, the CFTR-corrected C38 bronchial epithelial cell line, and the normal primary human bronchial epithelial cells, when ARSB was silenced, IL-8 secretion declined following exposure to TNF-α [29]. C4S content increased significantly, cell-bound IL-8 increased, and secreted IL-8 declined significantly. Cell fractionation demonstrated that the IL-8 content associated with the cell membranes was increased to twice that of the cytosolic fraction, and chemotaxis of neutrophils to the bronchial epithelial cells increased when ARSB activity was lower. Although the expression of IL-8 was not increased, the local inflammatory impact of IL-8 was increased due to sequestration by membrane C4S.

### 10.3. BMP4 Membrane Sequestration Increased when ARSB Is Reduced

In human colonic epithelial cells, when ARSB was inhibited by siRNA, the attachment of bone morphogenetic protein (BMP)-4 to the cell membrane increased and the expression of BMP4 by the epithelial cells decreased [30]. Exogenous BMP4 activated the phospho-Smad3 signaling pathway, leading to increased expression of CHST (carbohydrate sulfotransferase) 11, which leads to increased chondroitin 4-sulfate.

## 11. Effects on Proteoglycans and Chondroitin Sulfotransferases

Modification of N-acetylgalactosamine 4-sulfation by ARSB affects cell-cell signaling and cell-matrix interactions which are mediated by proteoglycans with C4S or dermatan sulfate attachments. Decline and overexpression of ARSB have been shown to modify the expression of protein components of proteoglycans linked with ARSB, as well as to modify the expression of chondroitin sulfotransferases.

### 11.1. Increased Expression of Syndecan-1, Decorin, Versican, CSPG4, and Neurocan

Expression of the protein components of the proteoglycans syndecan-1 and decorin was significantly up-regulated following overexpression of ARSB in mammary epithelial cells [94]. Soluble syndecan-1 secretion increased following increase in ARSB activity and decreased after silencing of ARSB activity by siRNA.

In prostate epithelial cells, decline in ARSB and the resulting increase in chondroitin 4-sulfation were associated with increased expression of versican [27]. Versican is a high MW proteoglycan with chondroitin sulfate and hyaluronan attachments and several EGF-like attachments at its carboxy-terminus.

In the human melanoma cell lines, expression of CSPG4 (chondroitin sulfate proteoglycan 4, also known as melanoma specific proteoglycan) increased following silencing of ARSB [33]. The increase was facilitated by the increased availability of galectin-3, when chondroitin 4-sulfation was increased. Galectin-3 silencing inhibited the ARSB siRNA-induced increase in CSPG4.

Increased expression of neurocan followed ARSB silencing and the resulting increased levels of sulfated GAG and C4S in cultured astrocytes from rat cortex and hippocampus [75]. Astrocyte-mediated neurite outgrowth was inhibited in co-cultures of rat hippocampal astrocytes and neurons when ARSB was silenced, and neurite outgrowth was stimulated by treatment with recombinant ARSB.

### 11.2. Reduced Carbohydrate Sulfotransferase (CHST)11 Expression

Decline in arylsulfatase B led to decline in CHST11 (carbohydrate sulfotransferase 11; chondroitin-4-sulfotransferase; C4ST) mRNA expression in human colonic epithelial cells and in colonic epithelium of ARSB-deficient mice [30]. The decline in CHST11 expression following ARSB reduction was attributed to effects of ARSB on bone morphogenetic protein (BMP) 4, since BMP4 expression and secretion declined when ARSB was silenced. When chondroitin 4-sulfate (C4S) was more sulfated due to decline in ARSB, more BMP4 was sequestered by C4S in the cell membrane, signaling through phospho-Smad3 was inhibited, and phospho-Smad3 binding to the CHST11 promoter was inhibited. This pathway suggests a possible feedback inhibition in which more C4S is not produced when BMP4 is sequestered with more highly sulfated C4S.

### 11.3. Increased Carbohydrate Sulfotransferase (CHST)15 Expression

In prostate stem cells, when ARSB was silenced, the expression of CHST15 (carbohydrate sulfotransferase 15; N-acetylgalactosamine 4-sulfate 6-O-sulfotransferase) increased [38]. The pathway leading to increase involved Rac-1 GTPase activation, phospho-p38 MAPK, and nuclear GATA-3. Increase in 4,6-disulfated chondroitin sulfate E disaccharides was demonstrated in ARSB-null mouse hepatic tissue by disaccharide analysis, consistent with an impact of ARSB decline on CHST15 expression.

## 12. Role in Prostate Development and Epithelial-Mesenchymal Identity/Transition

In cultured normal human prostate stromal and epithelial cells and in prostate stromal and epithelial cells obtained by laser-capture microdissection from normal and malignant human prostate tissues, ARSB expression was significantly greater in the stroma than in the epithelium [37]. This is consistent with findings in the developing rat ventral prostate tissue in which GALNS immunochemical intensity was greater in the epithelium than the stroma and ARSB intensity was greater in the stroma [71]. In prostate malignancy, mRNA expression of epithelial ARSB declined and expression of GALNS increased [37]. Markers of stroma, including vimentin, and markers of epithelium, including E-cadherin, reflected this distinction between epithelium and stroma.

When ARSB was silenced in cultured prostate epithelial cells, expression of CHST15 increased [38]. In association with increased GALNS of the malignant epithelium, the increase in CHST15 can lead to increased C4S, since C4S can increase in the epithelial cells by removal of 6-sulfate from chondroitin sulfate E (chondroitin-4,6-sulfate). Increase in C4S indicates an increased mesenchymal phenotype, such as detected in cultured human prostate stromal cells (~8.0 μg/mg protein), exceeding the value (~5.5 μg/mg protein) in the prostate epithelial cells [27].

Experimental findings indicate distinct variation in expression and activity of sulfatases, sulfated GAGs, C4S, and versican in the process of normal prostate development. Interference by estrogen in normal prostate development may be attributable, at least in part, to disruption of sulfatase activity. Immunohistochemistry of post-natal (days 1–30) ventral rat prostate with specific ARSB and GALNS antibodies demonstrated distinct and reciprocal localization of ARSB and GALNS [71]. ARSB immunostaining was predominant in the stroma, and GALNS was predominant in the epithelium.

## 13. Effect on Mitochondrial Metabolism and Mediation of Oxygen Signaling

### 13.1. Abnormal Mitochondria in MPS VI

Mitochondria in fibroblasts of MPS VI patients and in a rat model of MPS VI had reduced mitochondrial membrane potential and defective mitochondria [95]. These findings were associated with impaired autophagy and increased polyubiquitination in visceral organs of the MPS VI rats. ARSB had previously been detected in mitochondria of epithelial cells of the proximal convoluted tubules of the rat kidney [46]. By electron microscopy, we observed abnormalities of the mitochondria in the hepatic epithelium of ARSB-null mice, consistent with the observations in MPS VI patients [52]. Notably, the mitochondria were irregular with a central deposition of dark granules and the lamellae were irregular and disrupted. Some mitochondria were elongated, and mitochondrial membrane integrity was compromised. Autophagocytic vacuoles and lysosomal accumulation were evident near the damaged mitochondrial membranes.

### 13.2. Enhanced Aerobic Glycolysis (the Warburg Effect) with Decline in Mitochondrial Membrane Potential, Complex I Activity, and Oxygen Consumption Rate and with Increase in Extracellular Acidification Rate when ARSB Is Silenced

Mitochondrial membrane potential in ARSB-silenced HepG2 cells and in primary hepatocytes from ARSB-null mice was significantly decreased [52]. In the ARSB-null mice, the Mitochondrial Complex I activity was 45% less than control. In HepG2 cells, Complex I activity declined by 40% with respect to control when ARSB was silenced.

Mitochondrial function in the ARSB mice was studied by measurements of oxygen consumption rate (OCR) and extracellular acidification rate (ECAR). In HepG2 cells, silencing ARSB inhibited the OCR significantly within 30 min and OCR remained significantly reduced at 90 min. Treatment by FCCP, an uncoupler of oxidative phosphorylation, produced a significantly higher ECAR when ARSB was silenced in the HepG2 cells. Serum lactate concentration increased about 20% in ARSB-null mice, consistent with enhanced anaerobic metabolism. Pyruvate concentration in the mitochondria of the ARSB-null mouse liver was 35% lower than control. These experimental results suggested increase in aerobic glycolysis, i.e., the Warburg effect, when ARSB activity was diminished. We hypothesized that in the absence of ARSB activity, sulfate could not undergo reduction and was unavailable to interact with iron and the Fe-S clusters were disrupted. In the absence of ARSB activity, the cells were unable to optimally utilize available oxygen and glycolysis was increased.

### 13.3. Silencing ARSB Replicates Effects of Hypoxia in Human Bronchial Epithelial and Colonic Epithelial Cells

In studies of ARSB and hypoxia [35], the correlation r between the effects of ARSB silencing and exposure to 10% oxygen for 4 h on Ct values for expression of 84 genes in a hypoxia gene array was 0.994. Hypoxia reduced ARSB activity and increased total sulfated glycosaminoglycans and chondroitin 4-sulfate, as measured by the 1,9-dimethylmethylene blue Blyscan assay and specific chondroitin 4-sulfate antibody. ARSB silencing and overexpression had inverse effects on the expression of HIF-1α.

### 13.4. Impaired Production of Sulfhydryls and Reduced Glutathione

Total cellular and protein-associated sulfhydryls and GSH/GSSG (reduced glutathione/glutathione disulfide) ratios were significantly (*p* < 0.001) lower in human bronchial and intestinal epithelial cells following ARSB silencing or hypoxia, compared to control-silenced or normoxia [35]. Similarly, the reduced glutathione to glutathione disulfide ratio (GSH/GSSG) and the total cellular and protein-associated sulfhydryl concentrations were significantly less in ARSB-null mouse hepatic cells, compared to normal control. These findings demonstrate marked declines in the capacity for sulfate reduction in the ARSB-null mouse and following silencing of ARSB. However, overall reducing capacity was increased with declines in the NAD+/NADH and NADP+/NADPH ratios, due to increases in NADH and NADPH. Both NADH oxidase and NADPH oxidase activity were significantly reduced in the hepatic tissue of the ARSB-null mice, consistent with these lower ratios.

## 14. Diseases with Deficiency of Arylsulfatase B

The pathophysiology of the diseases initially associated with decline in ARSB (MPS VI and MSD) are attributed to the accumulation throughout tissues of the undegraded sulfated polysaccharides. Hydrolysis of the 4-sulfate group at the non-reducing end of chondroitin 4-sulfate and dermatan sulfate is required for subsequent chondroitin degradation [1,2,3,4,5]. Pathophysiology occurs due to abundance of unmetabolizable sulfated GAG. Subsequent investigation has extended recognition of molecular reactions activated or inhibited by the excessive 4-sulfation and relative over-abundance of the associated sulfated GAGs in human cells. Diseases associated with decline in ARSB are listed in Table 3.

### 14.1. Mucopolysaccharidosis VI

Clinical observations in the 1960’s identified the lysosomal storage disorder known as Maroteaux-Lamy-Syndrome (MLS), which was characterized by specific findings, including visceromegaly and corneal clouding due to accumulation of mucopolysaccharides [6,7,8,9,10,11]. Chondroitin sulfates were identified in the urine, and impaired catabolism, not over-production, became recognized as the cause of the accumulation of the mucopolysaccharides. Arylsulfatase B (ARSB) was first described in the mid-20th century when it was identified in human liver [130] and subsequently associated with MLS or Mucopolysaccharidosis (MPS) VI, as summarized by McKusick [131]. It was distinguished as a type II arylsulfatase with activity toward nitrocatechol sulphate (NCS) and distinct from Arylsulfatases A and C. Early studies on sulfatases were also performed by Dzialoszynski in 1947 and others [132,133,134,135,136].

Subsequently, deficiency of ARSB was detected in cultured fibroblasts of patients with the Maroteaux-Lamy Syndrome (MLS) [137], and the relationship between inherited deficiency of ARSB and MLS, subsequently identified as Mucopolysaccharidosis (MPS) VI, was clarified in several studies in the 1970s [6,7,8,9,133,136]. MPS VI was recently reviewed in detail [10], including information about the associated ARSB mutations identified worldwide. MPS occurs at a frequency of about 1/250,000–1/600,000 per live births, al-though incidence may be higher in some groups and the diagnosis may be missed [138]. Life expectancy is increasing with replacement therapy and varies based on age at diagnosis and disease severity, but generally is to the second or third decade of life. In 103 patients who received enzyme replacement therapy (ERT), mean age at death was 22.9 ± 11.4 years [139]. Specific mutations have been associated with varying degrees of disease severity, and baseline (at the time of initiation of ERT) urinary glycosaminoglycan levels <200 µg/mg creatinine are associated with better outcomes [10,139].

Many therapeutic efforts to correct ARSB deficiency have been initiated and have been reviewed [10]. Efforts included bone marrow transplantation, umbilical cord blood transplantation, enzyme replacement therapy, (Galsulfase, Naglazyme), and viral-mediated gene transfer [140,141,142,143]. These initiatives have developed from the detailed animal models of MPS VI [96,144,145,146,147].

### 14.2. Multiple Sulfatase Deficiency

The formylglycine modifying enzyme (FGE), converts the critical cysteine 91 residue in ARSB to C-formylglycine (FGly; 3-oxoalanine) to enable binding and hydrolysis of the 4-sulfate group of C4S and DS. Mutations of the sulfatase modifying factor (*SUMF*)-1 gene result in the disorder multiple sulfatase deficiency (MSD), since the family of eukaryotic sulfatase enzymes requires post-translational modification for activity [23,24,148,149,150,151,152]. The common mechanism whereby the FGE activates sulfatase has been detailed and shows the requirement for stabilization of the active site by several well-situated cationic residues [22,25]. Post-translational modification requires the conversion of cysteine 91 of ARSB located in the sequence PLCTPSRSQLLT to 3-oxoalanine (also known as C-formylglycine, FGly) [26]. This post-translational modification is severely defective in MSD, which includes metachromatic leukodystrophy, steroid sulfatase deficiency, X-linked dominant chondrodysplasia punctata, and MPS II, IIIA, and VI [150]. MSD has also been called Austin syndrome, or mucosulfatidosis. Manifestations predominantly involve brain, skin, and skeleton, and may include ichthyosis, hepatosplenomegaly, and cognitive impairment, and are classified as neonatal, late-infantile, and juvenile types, depending on age at diagnosis. MSD is considered as an ultra-rare disease and predicted life expectancy was reported as less than 2 years [153].

### 14.3. Cystic Fibrosis

Cystic fibrosis (CF) is an inborn genetic disorder caused by mutations in the cystic fibrosis transmembrane conductance regulator (*CFTR*) gene, which impair its function to regulate a chloride ion channel and fluid secretion across epithelial cell membranes [97]. The dominant clinical manifestation of CF is respiratory failure, due to accumulation of viscous secretions and chronic infection. Prior to identification of defective *CFTR* as the cause of CF, CF, also known as mucoviscoidosis, was considered among the lysosomal storage diseases [154,155]. In an early report, ARSB activity was noted to be reduced in lymphocytes from two CF patients [137]. Other studies showed oversulfation of glycoconjugates synthesized by CF epithelial cells of lung, pancreas, and other organs, increases in the glycosaminoglycans dermatan sulfate and chondroitin sulfate in skin fibroblasts of CF patients, and accumulation of sulfated polysaccharides in tissues and in urine of CF patients, as summarized in a review [154]. Significant progress has been made in life expectancy and quality of life with treatment directed at correction of CFTR [156]. Median life expectancy is reported to be 44 years in the United States [157]; disease prevalence is 1/3900 live births in the United States [158].

In a 2007 report, ARSB activity was compared in three pairs of human airway epithelial cells which had either functional CFTR or non-functional CFTR [39]. ARSB activity increased by 40% in the CFTR-corrected cells, with corresponding declines in chondroitin 4-sulfate content. Italian data using a blood spot screening assay showed decline in ARSB in 57 children with CF compared to 181 unaffected controls [58]. Clinical study of 16 CF patients demonstrated that ARSB activity was significantly less in circulating neutrophils and mononuclear cells than in similar cells from 31 control subjects [88]. In the plasma of the CF patients, Interleukin (IL)-6 was significantly increased, and IL-8 was reduced, compared to the normal controls.

Other in vitro studies showed that when CFTR was corrected by a small molecule potentiator (VRT-532, Vertex), which normalized CFTR function at the cell membrane, ARSB expression and activity increased to the level in the normal bronchial epithelial cells [89]. In contrast, ARSB expression and activity were unaffected by a CFTR corrector (VRT-534, Vertex), which improved CFTR migration to the cell membrane. Concomitantly, with treatment by the CFTR potentiator, total sulfated glycosaminoglycans and C4S declined, secreted IL-8 increased, secreted IL-6 declined, and neutrophil chemotaxis to the spent media obtained from the potentiator-treated CF cells increased. Other studies indicated that IL-8 attachment to human bronchial epithelial cells increased when ARSB was silenced by siRNA, due to increased binding of IL-8 with the more highly sulfated chondroitin 4-sulfate present on the surface of the cultured cells [29]. Consequently, leukocyte chemotaxis to the epithelial cells increased due to epithelial cell sequestration of IL-8. In addition, decline in ARSB activity led to increased expression of GPNMB (glycoprotein transmembrane nonmetastatic melanoma protein B) in hepatic tissue of ARSB-null mice and to increased levels in plasma and in circulating leukocytes from CF patients and cultured CF bronchial epithelial cells [83]. These effects indicate the impact of decline in ARSB and the resulting increase in C4S on inflammatory processes which contribute to the pathophysiology of CF. Publications have suggested that CF carrier advantage status may provide protection from tuberculosis and malaria [159,160]. Decline in ARSB and change in chondroitin sulfation have been considered in relationship to protection from these diseases [76,77,78,79,97].

### 14.4. Malignancy

#### 14.4.1. Overall Impact on Molecular Pathways Contributing to Malignancy and Role as Tumor Suppressor

ARSB was significantly lower in malignant tissue and malignant cell lines from colon, prostate, mammary, and melanoma than control samples [33,34,41,42,43,72,94]. Multiple mechanisms may contribute to the impact of decline in ARSB on propensity to malignancy. These mechanisms are considered in relationship to malignancy in this section. Transcriptional effects on genes involved in activation of Wnt signaling and in proteoglycan expression are mediated through changes in binding of galectin-3 and SHP2 to chondroitin 4-sulfate when it is more or less sulfated, depending on ARSB activity. Decline in ARSB leads to reduced binding of galectin-3 with C4S and increased availability of galectin-3 for nuclear translocation and cooperation with AP-1 and Sp1 in promoter activation, for expression of genes such as Wnt9A in colonic epithelium [32], versican in prostate cells [27], and CSPG4 in melanoma [33]. In contrast, SHP2 binds more tightly with more highly sulfated C4S when ARSB is reduced, leading to sustained ERK1/2, JNK, or p38-MAPK phosphorylation and increased expression of pro-MMP-2 in melanoma [33], EGFR in prostate [28], CHST15 (carbohydrate sulfotransferase 15; N-acetylgalactosamine 4-sulfate 6-O-sulfotransferase) in prostate [38], MITF (melanocyte inducing transcription factor) in liver [14], and enhanced promoter methylation with decline in expression of DKK3 (Dickkopf Wnt signaling pathway inhibitor) in prostate [36,37]. The impact of decline in ARSB on mitochondrial disruption and potential for enhanced aerobic glycolysis was presented and also considered in relation to replication of effects of hypoxia by decline in ARSB [35,52].

The limited life expectancy of individuals with MPS VI or MSD and the low incidence of these diseases make it unlikely that diseases prevalent at older ages, such as cancer, cardiovascular disease, or diabetes would be detected at increased frequency in MPS VI or MSD. Notably, CF patients, who are achieving longer survival, have increased incidence of several malignancies, particularly of digestive organs [161,162,163].

#### 14.4.2. Decline in ARSB in Colon Malignancy

Decades ago, early investigators of sulfatases examined sulfatases in colon malignancies, but studies were limited due to limited investigative tools [132,164]. With improved immunhistochemical techniques, antibodies, and tissue microarrays, recent studies have shown that the intensity of ARSB immunostaining was reduced in higher grade colonic adenocarcinomas [41]. The ARSB staining intensity and localization differed in malignant colonic tissue from normal colonic epithelium, with distinctive, intense luminal membrane staining reduced in the malignancies and less in the grade 3 than in the grade 1 adenocarcinomas. In malignant colonic tissue, the ARSB activity was significantly lower than in the normal control tissue. In addition, in cultured metastatic colonic epithelial cells (T84 cells), the ARSB activity was lower than in normal control cells, and the decline in ARSB was associated with increased expression of MMP-9, RhoA activity, and enhanced cell migration [34]. These effects were reversed in the malignant cells by ARSB overexpression. In the human colonic epithelial cells, when ARSB activity was reduced, expression of Wnt9A increased, whereas expression of BMP4 and carbohydrate sulfotransferase (CHST)11 declined [30,32]. The activation of Wnt-β-catenin pathway is recognized as a critical pathway in colon carcinogenesis [165] and was enhanced when ARSB was reduced. Decline in circulating RNA of ARSB was reported in 45 consecutive patients with colorectal cancers, compared to healthy controls [98].

#### 14.4.3. Decline in ARSB in Prostate Malignancy

Increased C4S and increased abundance of the chondroitin sulfate proteoglycan versican were previously reported by other investigators as biomarkers of more aggressive prostate cancer [99,100,101]. Subsequently, analysis of ARSB immunochemistry in prostate cancer tissue microarrays including nearly 300 cases showed that lower ARSB intensity scores were associated with higher Gleason scores and with increase in biochemical recurrences [42,43]. Decline in ARSB was associated with increased C4S and increased expression of versican in prostate cells and tissue [27]. The mechanism of increased versican expression was attributable to reduced binding of the galactoside-binding protein galectin-3 with more highly sulfated chondroitin 4-sulfate present when ARSB is reduced. Reduced binding leads to increased availability for circulating and nuclear translocation of galectin-3 and interaction with nuclear AP-1 to activate the versican promoter in prostate cells. Inversely, binding with C4S of the ubiquitous tyrosine phosphatase SHP2 (PTPN11) increased in prostate cells when ARSB declined and chondroitin-4 sulfation was increased, leading to enhanced expression of EGFR through effects on phospho-JNK [28]. Treatment with exogenous EGF enhanced BrdU incorporation following silencing of ARSB, compared to normal controls [28].

Activation of Wnt/β-catenin signaling occurred in prostate epithelial cells [36,37] due to disinhibition of Wnt signaling when the expression of Dickkopf Wnt Signaling Pathway Inhibitor (DKK)3, which normally inhibits binding of Wnt with the Frizzled/LRP5/6 receptor complex, declined. DKK3 expression declined following methylation of the DKK3 promoter, when SHP2 was inhibited by increased chondroitin 4-sulfation, leading to increased phospho-ERK mediated increases in c-Myc/Max DNA binding, DNA methyltransferase (DNMT) activity and expression of DNMT 1 and 3a in prostate cells. Other experiments showed decline in DKK3 expression in tissue from prostate cancers and in prostate stem cells following ARSB silencing. Exposure of human prostate epithelial cells to dihydrotestosterone reduced ARSB and DKK3 expression, indicating androgen effect on ARSB and Wnt signaling [37]. Reduction of ARSB induced the hypermethylation of the DKK3 promoter and inhibited the DKK3 expression in prostate epithelial cells. Signaling by Wnt3A expressed by prostate stromal cells was facilitated by decline in epithelial DKK3, and expression of Wnt dependent genes, including c-Myc, GATA3, and Cyclin D1, increased in the epithelial cells. These observations indicate how ARSB-mediated effects contribute to Wnt signaling and prostate stem cell growth and malignant transformation.

In cultured human prostate stem and epithelial cells, inverse effects of ARSB and N-acetylgalactosamine-6-sulfate sulfatase (GALNS) on activation of Wnt signaling occur [37]. GALNS is the enzyme that removes 6-sulfate groups from chondroitin 6-sulfate and is required for the degradation of chondroitin 6-sulfate (C6S) and keratan sulfate. In vitro experiments showed that C6S can inhibit the binding of SHP2 with C4S [28], and both decline in ARSB and increase in GALNS activate Wnt signaling by increased methylation of the DKK3 promoter in the prostate stem cells due to effects on availability of phospho-SHP2 [36]. Experiments have also shown that expression of ARSB is greater in the normal prostate stromal cells and that expression of GALNS is greater in the normal prostate epithelial cells than in the stromal cells [27,37]. In the malignant prostate epithelial cells, GALNS activity increased. Expression of carbohydrate sulfotransferase (CHST)15 also increased in the malignant prostate epithelium and involved p38-MAPK pathway activation [38].

#### 14.4.4. Decline in ARSB in Mammary Malignancy

In malignant mammary tissue, the ARSB activity was reduced compared to normal [94]. This was consistent with previous findings that ARSB activity was significantly less in malignant mammary cell lines than in normal mammary epithelial and myoepithelial cells [72].

#### 14.4.5. Decline in ARSB in Malignant Melanoma

In human malignant melanoma cell lines, ARSB activity declined progressively with increased invasiveness of the melanoma cell lines [33]. Correspondingly, the chondroitin 4-sulfate increased, as ARSB declined from normal melanocytes to metastatic melanoma cells. These changes were associated with increased expression of CSPG4, the proteoglycan known to be increased in melanoma, through a galectin-3/Sp1 transcriptional mechanism. In addition, increased pro-MMP2 expression was mediated by increased binding of the non-receptor tyrosine phosphatase SHP2 to C4S, leading to increased phospho-ERK1/2. The combined effects of decline of ARSB and increase of C4S, CSPG4, and MMP2 significantly increased the invasiveness of the melanoma cells. Treatment with rhARSB reversed the observed increase in invasiveness.

Other studies have identified increase in programmed death ligand 1 (PD-L1) following decline in ARSB by siRNA in the cultured melanoma cells and in melanoma tissue with reduced ARSB [85]. Recent studies (unpublished) demonstrate that treatment with rhARSB retards the growth of cutaneous B16F10 melanomas in the C57/BL/6J mouse and reduces the expression of PD-L1. BrdU incorporation was inhibited when murine malignant melanoma cells were treated with rhARSB (upublished).

#### 14.4.6. Reduced ARSB Activity in Hepatic Carcinoma

In human hepatic carcinoma tissue and in the malignant hepatic cell line HepG2, ARSB activity was reduced compared to normal control, and the expression of Glycoprotein Nonmetastatic Melanoma Protein B (GPNMB) was increased in hepatic tissue of ARSB null mice and ARSB silenced hepatic cells [14]. GPNMB expression is known to correlate very highly with the invasive and metastatic characteristics of several malignant tissues and is a therapeutic target [90,91]. GPNMB expression in the hepatic cells was regulated by the transcription factor microphthalmia-associated transcription factor (MITF).

#### 14.4.7. Association of Thyroid Cancer and ARSB

In a genome-wide association study (GWAS) of differentiated thyroid cancer in an Italian population, an ARSB SNP was identified and associated with occurrence of thyroid cancer [102]. The SNP rs13184587 was in the intronic region of ARSB on chromosome 5. The effect was observed in two distinct Italian cohorts, with a total of 2075 cases and 1955 controls with a *p*-value of 8.54 × 10^−6^. However, this relationship was not confirmed in two smaller test populations.

#### 14.4.8. Other Findings

Some early studies from previous decades showed dissimilar results to the above findings and did not report lower ARSB in association with malignancy. Discrepancies may be attributable to non-specific antibodies or to assays that did not accurately detect ARSB activity or distinguish between ARSB and ARSA [132,166]. Elevated Arylsulfatase B activity was detected in 71% of 24-h urine samples from 243 patients with colorectal cancer [164], but in studies of arylsulfatase in lung, liver, and kidney, the assay used did not distinguish ARSB from ARSA or ARSC (steroid sulfatase) [167].

An acidic variant of ARSB (B1) which was phosphorylated on its protein and carbohydrate moieties, was identified from transplantable human lung cancer tissues [168]. This variant B1 and a cAMP-dependent protein kinase responsible for phosphorylation of arylsulfatase B were identified in the lung tumor tissue. Type II isozyme activity was found to be elevated and considered responsible for the over-phosphorylation of arylsulfatase B and reduced ARSB activity. A recent review has included reports about sulfatases in normal and malignant tissues [169].

### 14.5. Neurological Disorders

Decline in ARSB and the resulting increase in chondroitin 4-sulfate have been investigated in a wide range of neurological disorders, including spinal cord injury, peripheral nerve injury, optic nerve injury, traumatic brain injury, and Alzheimer’s Disease.

#### 14.5.1. Spinal Cord Injury

Treatment with recombinant ARSB improved recovery from spinal cord damage in a mouse model of spinal cord injury [104]. Underlying pathology demonstrated the increased accumulation of chondroitin sulfate at the site of injury. Recombinant human ARSB was reported to improve functional recovery and eliminated the observed immunoreactivity for chondroitin sulfates within five days. In other experiments, a hydrogel, which was an imidazole-polyorganophosphazene-hydrogen complex with sustained release of ARSB, significantly diminished fibrotic extracellular matrix components associated with spinal cord injury and improved functional locomotor recovery with an increased number of regenerating axons [105]. In contrast, chondroitin 6-sulfatase did not influence the extent of axon regeneration.

#### 14.5.2. Peripheral Nerve Injury

Other studies reported the effectiveness of rhARSB in repair of damaged nerves in animal models [106,107]. The underlying pathophysiology demonstrated the buildup of chondroitin 4-sufate at the site of the lesion and the interference with restoration of nerve pathways. Recombinant ARSB was effective in reducing the inhibition by chondroitin sulfate proteoglycans (CSPGs) in in vitro models of the glial scar and after crush nerve injury in adult mice. The chondroitin 4-sulfation was recognized as the critically important sulfation. Similar effects of CSPGs were detected after optic nerve injury in rodent models [108].

Exogenous ARSB was observed to enhance neurite outgrowth following ethanol-induced injury [75]. Astrocyte ARSB declined and C4S increased following ethanol exposure which impaired neurite outgrowth. Expression and distribution of ARSB were associated with neuronal death in a superoxide dismutase (SOD)1 transgenic mouse model [109].

#### 14.5.3. Traumatic Brain Injury (TBI)

Increases in chondroitin 4-sulfate (C4S) and chondroitin sulfate proteoglycans (CSPGs), including neurocan, have been identified and recognized as major contributors to the scar formation that follows traumatic brain injury [110]. Post-traumatic decline in ARSB contributes to the accumulation of C4S in traumatic brain injury (TBI) due to inhibition of C4S degradation. Studies of traumatized and control brain indicated that the overall increase in C4S resulted from both decline in ARSB, leading to inhibition of C4S degradation, and increased carbohydrate sulfotransferase (CHST)11 and sulfotransferase activity, leading to increased synthesis of C4S.

In primary astrocyte culture, ASRB activity was decreased post injury induction by scratch [110]. Initial studies of ARSB in human brain were performed decades ago [49,50].

#### 14.5.4. Alzheimer’s Disease

A genome wide association study (GWAS) with 381 participants (172 cases, 209 controls) in the Alzheimer’s Disease Neuroimaging Initiative identified 21 genes or chromosomal areas with at least one Single Nucleotide Polymorphism (SNP) that was considered as a potential new candidate for occurrence of sporadic AD [111]. *ARSB* (SNP rs337847, intron 3) had a *p*-value of 6.71 × 10^−6^ in association with increased occurrence of AD.

Other data have shown an association between ARSB and survival of neurons in brains affected by AD [112]. The abundance of amyloid beta peptide (A beta) and the selective loss of neurons characteristic of Alzheimer’s disease were also characterized by survival of subpopulations of brain cells. The gene expression profiles of the surviving neurons were examined for characteristics enabling resistance to A beta toxicity. By a differential display technique used to compare profiles of gene expression in an amyloid beta peptide-resistant cell line with its parental cells, increased expression of two components of the endosomal-lysosomal system, ARSB and insulin growth factor II receptor/mannose-6-phosphate receptor, was detected in the amyloid beta peptide-resistant population [112]. In the cortex and hippocampus of ARSB null mice and control C57BL/6J mice, SSA protein was increased five-fold in the ARSB-null mice (unpublished data).

### 14.6. Association of ARSB with Infections

Changes in ARSB activity and chondroitin 4-sulfation have been recognized as factors affecting infectivity and disease progression.

#### 14.6.1. Malaria

Multiple studies have reported that attachment of malarial parasites to endothelium is less when chondroitin 4-sulfate is more sulfated [76,77,78,79,84]. This impact on binding affects infectivity in multiple organs, including placenta, lung, and brain. Experiments showed that chloroquine, an effective treatment for malaria, lowers ARSB activity and expression in human placental and cerebrovascular cells [44]. This is consistent with a mechanism in which decline in ARSB and the associated increase in chondroitin 4-sulfate inhibits attachment of malarial parasites.

#### 14.6.2. SARS-CoV-2

Recently, ARSB was shown by immunohistochemistry to be reduced in pulmonary sections from autopsy tissue of patients with COVID-19 [114]. Subsequent studies demonstrate that exposure of cultured human bronchial epithelial cells to the SARS-CoV-2 spike protein binding domain leads to decline in ARSB expression and activity (unpublished data).

#### 14.6.3. Parasitic Infections

Changes in ARSB were evaluated during treatment of Bancroftian filariasis [115]. Findings showed sequential changes in the eosinophil content of ARSB during treatment. The level of hepatic ARSB in schistosomiasis was reported to show changes in activity of the enzyme during the course of the infection [116].

### 14.7. Bone and Cartilage Disease

Primary manifestations of ARSB deficiency were noted in bone and cartilage when MPS VI was first described. Subsequent studies have further examined these manifestations. ARSB has been reported to be involved with skeletal turnover and noted to be increased [117]. Other experiments showed higher ARSB activity in cultured human chondrocytes from osteoarthritis [120]. Studies in cartilage have included co-culture of normal chondrocytes with chondrocytes from animal models of MPS VI [118,119]. Articular chondrocytes from affected animals had a high rate of apoptosis and released nitric oxide and inflammatory cytokines, suggesting a possible mechanism underlying chondrocyte cell death and degenerative joint disease in the mucopolysaccharidoses. Changes in the metabolism of chondroitin sulfate glycosaminoglycans in articular cartilage from patients have been identified in Kashin-Beck disease, an osteochondropathy endemic to China and nearby areas [47].

### 14.8. Pulmonary Disease

The effect of long-term oxygen therapy in patients with moderate COPD varied based on ARSB expression and genotype [122]. Changes in ARSB gene expression involving coding and non-coding regions were identified in patients with moderate COPD who were refractory to oxygen therapy in the Long-term Oxygen Treatment Trial (LOTT). Investigators found no benefit of long-term oxygen therapy in many patients with moderate chronic obstructive lung disease (COPD). In 331 subjects, 97 single nucleotide polymorphisms (SNPs) showed evidence of interaction with oxygen therapy at *p* < 1× 10^−5^, including 7 SNPs near Arylsulfatase B (ARSB; *p* = 6 × 10^−6^). In microarray expression profiling on 51 whole blood samples from 37 individuals, at screening and/or at 12-month follow-up, ARSB expression was associated with the primary outcome depending on oxygen treatment.

In the study of ARSB activity in peripheral leukocytes of cystic fibrosis patients 854, the subset of the controls with a diagnosis of asthma had reduced activity of neutrophil ARSB activity, compared to non-diseased controls. The human MPS VI endothelial cell model presented as a phenotype of pulmonary hypertension, which is present in human MPS VI disease. Increasing concentrations of dermatan sulfate were associated with reduction of eNOS expression and viability [121].

### 14.9. Cardiovascular Disease

Cardiac abnormalities are present in MPS VI patients and were noted in rodent models of MPS VI [6,7,8,9,10,11,123,124].

#### 14.9.1. Cardiac Disease

The microRNA, miR-154-5p, which acts to inhibit expression of ARSB, was identified as a regulator of angiotensin II-mediated heart remodeling [126]. The use of rhARSB was evaluated in heart failure in non-mucopolysaccharidosis failing hearts, but with increased chondroitin sulfate [125]. Rat left ventricles and failing human hearts were studied. Treatment with rhARSB in the transverse aortic constriction model of heart failure improved recovery. CHST15 was reported to be increased in a model a heart failure, and treatment with rhARSB was beneficial [127]. Sympathetic nerve regeneration was reported to be regulated by chondroitin sulfation following myocardial infarction [113].

#### 14.9.2. Hypertension

Investigation of sulfatase activity in salt-sensitive rats on high and low salt diets demonstrated that ARSB activity was higher in renal epithelium from the rats on the low salt diets than from rats on the high salt diet or from spontaneously hypertensive rats [31,70]. These findings are consistent with identification of several QTLs associated with ARSB in the rat genome database (Chromosome 2: 24067560-24228321) and linked with blood pressure, increased kidney mass, and increased cardiac mass.

#### 14.9.3. Vascular Disease

Increased ARSB was reported in varicose veins and varicose veins complicated by thrombophlebitis [128]. One study reported upregulation of ARSB (1.15-fold) in symptomatic patients with carotid atherosclerosis who had symptoms of cerebral embolization [129].

### 14.10. Diabetes

In a carrageenan-induced mouse model of diabetes, mice showed abnormal glucose tolerance after six days. Associated with increased fasting blood sugars, ARSB activity was reduced in the hepatic tissue of the carrageenan-exposed mice [80]. Decline in ARSB was associated with increase in circulating galectin-3 and increased binding of galectin-3 with the insulin receptor of the hepatic cells. In mouse studies, galectin-3 bound with the insulin receptor and inhibited downstream signaling from the insulin receptor [81]. Galectin-3 binding blocked the ability of the insulin receptor to combine with circulating insulin, leading to impaired insulin signaling, insulin resistance and decline in phospho-Akt1. In adult subjects with pre-diabetes, defined as hemoglobin A1c of 5.7–6.4%, ARSB activity increased in the subjects on a no-carrageenan diet, compared to subjects on a carrageenan-containing diet [82]. The lower ARSB levels in circulating leukocytes were associated with higher circulating galectin-3 and with higher hemoglobin A1c levels.

### 14.11. Other Pathologies Associated with ARSB

Other reports have identified pathophysiological effects of decline or increase in ARSB activity in association with: uterine leiomyomas [increased ARSB in small (<10 g), decreased in large (>100 g) [103]; increased serum ARSB activity in bullous pemphigoid [170]; increased ARSB activity in Wharton’s jelly from pre-eclampsia samples [171]; increased serum ARSB in nasal allergy, but reduced in severe obstruction [172]; reduced ARSB in lymphocytes from chronic lymphocytic leukemia [173]; and increased ARSB and a phosphorylated form of ARSB in peripheral leukocytes in chronic myelogenous leukemia [174].

## 15. Conclusions and Application

### 15.1. Not just a Lysosomal Enzyme—Membrane Localization of ARSB

**Figure 6:**
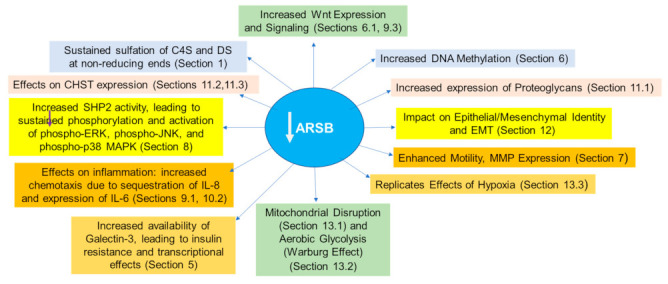
Predominant effects of decline in Arylsulfatase B (ARSB) in mammalian cells. Decline in ARSB leads to profound effects on vital cell processes in mammalian cells, as indicated in this report. C4S = chondroitin 4-sulfate; DS = dermatan sulfate; EMT = epithelial-mesenchymal transition; CHST = chondroitin sulfotransferase; ERK = extracellular-signal regulated kinase; MAPK = mitogen- activated protein kinase; JNK = c-Jun N-terminal kinase; SHP2 = tyrosine-protein phosphatase non-receptor type 11.

Arylsulfatase B was initially recognized as only a lysosomal enzyme, but recent studies demonstrate extra-lysosomal distribution and activity [39,40,41,42,43,44]. Decrease in activity increased the content of chondroitin 4-sulfate and total sulfated glycosaminoglycans. These increases were shown by measurements using specific C4S antibody and sulfated GAG assay and disaccharide analysis. The natural physiological substrate, chondroitin 4-sulfate, was present intracellularly, including in the nucleus, and in the extracellular matrix. Figure 6 presents the overall predominant effects of decline in ARSB in mammalian cells.

### 15.2. Impact of ARSB on Intracellular Signaling, Cell-Cell Signaling, and Transcription

The change in sulfation pattern of chondroitin 4-sulfate by inhibition or increase in ARSB has provided new insight into intracellular signaling and interactions between cells and with the extracellular matrix. Increased chondroitin 4-sulfation elicits signals to modulate vital cellular process, including transcriptional events. Effects include: (1) reduced binding of galectin-3 and increased availability for interaction with transcription factors and other mediators, including the insulin receptor [27,32,33,35,80,81,82]; (2) increased binding of SHP2 and inactivation of phosphatase activity [14,28,33,36,37,38]; (3) expression of proliferative genes, including *Wnt9A, c-Myc, EGFR, Cyclin D1, GATA-3* [28,32,35,37,38]; (4) activation and expression of matrix degradation genes, *pro-MMP2* and *MMP9* [33,34,83]; (5) sequestration and reduced secretion of critical molecules, including IL-8, high molecular weight kininogen, and BMP4 [29,30,31]; and (6) hypermethylation of the *DKK3* promoter and activation of Wnt signaling [36,37]. These effects influence cell migration and invasion and mediate cell proliferation by growth factors, thereby contributing to malignant transformation. Decline in SHP2 leads to sustained activation of p38MAPK, JNK and ERK1/2 [14,28,36], thereby impacting on critical phosphorylations and pathways of intracellular signal transduction. Increased availability of galectin-3 affects transcriptional events [27,35] and may, by binding with the insulin receptor, impair insulin signaling and associated downstream metabolic processes. This combined effect, of decline in SHP2-mediated phosphatase action and of increased galectin-3 on transcription and metabolism, enables ARSB to broadly regulate aspects of cell fate.

### 15.3. Mimicry of Effects of Hypoxia and Impact on Mitochondrial Structure and Function

Interestingly, and unexpectedly, decline in ARSB mimicked effects of hypoxia [35] and induced aerobic glycolysis [52], the Warburg effect, further substantiating a profound role of decline in ARSB on fundamental cellular metabolism and malignant transformation. 

### 15.4. Impact on EMT and Epithelial-Mesenchymal Identity

Recognition that ARSB and C4S predominate in the stroma and GALNS and C6S predominate in the epithelium [37,71] suggests a fundamental patterning which enables tissue organization due to particular spatial orientation and localization of sulfate groups. Interactions with specific chondroitin sulfations can program different responses to environmental cues.

### 15.5. Future Directions

Future work will clarify the distinct effects attributable to accumulation of chondroitin 4-sulfate and/or dermatan sulfate, including effects attributable to availability of more or less sulfated chondroitin or dermatan sulfate and effects due to specific, binding of critical molecules with the 4-sulfated GAGs. Elucidation of the impact of reduced availability of sulfate ion on cellular metabolism may provide new insight into fundamental cell biochemistry. 

Changes in cellular metabolism and cell signaling may accumulate over time when ARSB declines, perhaps due to impaired tissue oxygenation. Specific environmental triggers, such as hypoxia or increased chloride or phosphate in a microenvironment, may trigger decline in ARSB and subsequent alterations in cell signaling due to accumulation of C4S. Such changes may produce different effects in different tissues, leading to varied manifestations and evolution to different outcomes, such as malignancy vs. fibrosis. Although not as drastic as the changes seen due to congenital deficiency of ARSB, the compilation in time of significant cellular and tissue pathophysiology may affect vital organs, leading in time to organ failure or malignancy. Since ARSB requires post-translational modification for activation, identification of reduced expression of ARSB or of mutations of ARSB in gene reference databases may not capture significant changes in ARSB activity. Small, gradual increments in chondroitin 4-sulfate also may not come to attention, although they may lead to critical changes in cell signaling and even to epithelial-mesenchymal transition (EMT), as suggested in prostate cells and tissue.

Availability of specific antibodies to GAG chains has limited progress in the study of chondroitin sulfates. Previously, antibodies to C4S GAG chains were available, but are no longer commercially available and are difficult to produce. The chondroitin sulfate antibodies which detect the chondroitin stubs following treatment by chondroitinase ABC do not give information about C4S content. Heterogeneity of the chondroitin sulfate chains attached in proteoglycans is likely. Silencing ARSB and maintenance, thereby, of the 4-sulfate group at the non-reducing end of the GAG chain defines a distinct, mechanistic focus, as detailed in this report. This site appears to be critically important in regulation of vital cell processes. Disaccharide analysis provides quantitative assessment of the relative abundance of specific chondroitin disaccharides which populate the chondroitin sulfate chains, although providing no specific information about GAG chain length or variability.

Many of the reported studies with silencing of ARSB reduce ARSB expression to levels lower than those measured in biological samples. However, the underlying premise is that the mechanisms affecting transcription and cell signaling when ARSB is reduced and chondroitin 4-sulfation is increased are expected in localized, microdomains which form a nidus for cellular transformation and proliferation. The precise molecular interactions and the relative importance of declines in ARSB activity with respect to oncogenes and their mutations require further investigation. At present, data indicate that ARSB is a tumor suppressor gene and decline in ARSB can contribute significantly to malignant transformation. Future focus on ARSB and chondroitin sulfates is anticipated to provide useful information and approaches to human disease and may yield profound insight into fundamental biological processes.

## Figures and Tables

**Table 1:** The fluorometric plate assay for ARSB uses cell homogenate, not cell lysate. Units are expressed as nmol/mg protein/h [39,54].

Method for Measurement of ARSB Activity by Fluorometric Microplate Assay with 4-Methylumbelliferyl Sulfate	
**1**	The substrate is 5 mM of 4-methylumbelliferyl sulfate (4-MUS) in assay buffer, made fresh.
**2**	The assay buffer is 0.05 M Na acetate with 20 mM barium acetate, pH 5.6, at 37 °C.
**3**	Cell homogenate is prepared in ddH_2_O on ice, by sonication with metal tip, three times for 10 s.
**4**	20 µL cell homogenate is combined with 80 μL assay buffer and 100 μL substrate in wells of a microplate.
**5**	The microplate is incubated for 30 min at 37 °C.
**6**	The reaction is stopped by 150 μL of stop buffer (glycine 350 mM–carbonate 440 mM, pH 10.7).
**7**	Fluorescence is measured at 360 nm (excitation) and 465 nm (emission).
**8**	Enzymatic activity is expressed as nmol/mg protein/h using the protein content of the cell extract and a standard curve of 4-methylumbelliferone (MU) of known concentration.
**Reagents**	4-Methylumbelliferyl sulfate potassium salt (4-MUS; MW 294.32; C_10_H_7_KO_6_S); Na-acetate (MW 82.03; CH_3_COONa); Glycine (MW 75.07; NH_2_CH_2_COOH); Carbonate (MW 124.00; Na_2_CO_3_); Barium acetate [MW 255.42; (CH_3_COO)_2_Ba]
**Assay Buffer**	Na-acetate buffer 0.05 M with barium acetate 20 mM; pH 5.6
**Substrate**	5 mM 4-MUS substrate in Na-acetate/barium acetate buffer (fresh)
**Glycine-carbonate** **stop buffer**	Glycine 350 mM/Carbonate 440 mM; pH 10.7

**Table 2:** Inhibitors of Arylsulfatase B are presented in Section 4. Effects of other metals have been tested, revealing that barium had no impact on ARSB, but inhibited ARSA [65].

Inhibitors of Arylsulfatase B		
Substance by Category	Text Section	Reference
Ions:	4.1	
Chloride		[64,65,66,70]
Phosphate		[39,63,67,68]
Sulfate		[64,67,68]
Sulfite		[64,67,68]
Metals:	4.2	
Vanadium		[15]
Hormones:	4.3	
Estradiol		[71,72]
Estrone		[72]
Estradiol sulfate		[72]
Estrone 3-sulfate		[72]
Dihydrotestosterone		[37]
Chemicals:	4.4	
Ascorbic Acid		[69]
Carrageenan		[73,74]
Chloroquine		[44]
Ethanol		[75]
Other:	4.5	
Hypoxia		[35]

**Table 3:** Diseases with known association with ARSB or with chondroitin 4-sulfate are presented. Some reports present data about chondroitin 4-sulfate without ARSB data.

Diseases Associated with Decline in ARSB or with Increase in Chondroitin 4-Sulfate			
Disease	Mechanism/Etiology	Text Section	Reference
Mucopolysaccharidosis VI (MPS VI; Maroteaux-Lamy Syndrome)	Congenital mutations in ARSB	14.1	[6,7,8,9,10,11]
Multiple Sulfatase Deficiency (MSD)	Congenital mutations of SUMF	14.2	[96]
Cystic Fibrosis	Congenital mutations in CFTR	14.3	[39,58,83,88,89,97]
Malignancy	Acquired decline in ARSB expression or activity	14.4	
Colon	Increased Wnt9A	14.4.1	[30,32,34,41,98]
Prostate	Increased Wnt3A due to disinhibition of Wnt signaling; inhibition of ARSB by hormones	14.4.2	[27,28,36,37,38,42,43,71,99,100,101]
Mammary	Inhibition of ARSB by estrogen	14.4.3	[72,94]
Melanoma	Inhibition of ARSB and decline in PD-L1	14.4.4	[33,85]
Liver	Increased GPNMB and MITF	14.4.5	[14]
Thyroid	SNP in ARSB intron; GWAS study	14.4.6	[102]
Uterine Leiomyoma	Increased ARSB in small; reduced ARSB in large	14.11	[103]
Neurological Disorders		14.5	[50,104,105,106,107,108,109,110,111,112]
Spinal Cord Injury	Treatment with recombinant ARSB; identification of increased C4S	14.5.1	[104,107]
Nerve injury	Treatment with recombinant ARSB	14.5.2	[107]
Optic nerve injury	Treatment with recombinant ARSB	14.5.2	[106,108]
Ethanol-induced nerve injury	Inhibition of ARSB; treatment with recombinant ARSB	14.5.2	[75]
Traumatic brain injury	Inhibition of ARSB by hypoxia	14.5.3	[110]
Alzheimer’s disease	SNP in ARSB intron; Higher ARSB expression in amyloid beta-peptide resistant neurons	14.5.4	[111,112]
Sympathetic nerve regeneration post myocardial infarction	Treatment by recombinant ARSB	14.9.1	[113]
Infection		14.6	[76,77,78,79,114,115,116]
Malaria	Lower ARSB, higher C4S associated with resistance to infection	14.6.1	[76,77,78,79]
COVID-19	Lower ARSB and increased chondroitin sulfate by imaging	14.6.2	[114]
Parasitic disease: Filariasis Schistosomiasis	ARSB reduced then increased in eosinophils during and after treatment; Increased hepatic ARSB	14.6.3	[115,116]
Bone and Cartilage Disease	Characteristic of MPS VI with low ARSB	14.7	[6,7,8,9,10,11,117,118,119]
Osteoarthritis	Higher ARSB intracellularly and secreted from cultured chondrocytes of osteoarthritis	14.7	[120]
Kashin-Beck disease	Higher ARSB immunostaining	14.7	[47]
Pulmonary Disease	Characteristic of MPS VI with low ARSB	14.8	[6,7,8,9,10,11,121]
Asthma	Low ARSB in circulating leukocytes	14.8	[88]
COPD unresponsive to oxygen	ARSB expression and ARSB-associated SNPs in relation to response to oxygen	14.8	[122]
Cardiovascular Disease	Characteristic of MPS VI with low ARSB	14.9	[6,7,8,9,10,11,123,124]
Heart Failure	Treatment with recombinant ARSB in rat model	14.9.1	[125]
Angiotensin II-mediated	Treatment with miR-154-5p to inhibit ARSB expression in mouse model	14.9.1	[126]
Experimental Autoimmune Myocarditis	Inhibit CHST15 with siRNA	14.9.1	[127]
Hypertension	Lower ARSB with high salt diet	14.9.2	[31,70]
Varicose veins and varicose veins with thrombophlebitis	ARSB increased	14.9.3	[128]
Carotid atherosclerosis more likely to embolize	ARSB expression increased 1.15 times	14.9.3	[129]
Diabetes	Reduced ARSB in leukocytes	14.10	[80,81,82]

## Data Availability

Data are available through communication with the authors.

## Abbreviations

AP-1	activating protein-1 transcription factor
ARSA	arylsulfatase A
ARSB	arylsulfatase B
ATCC	American Type Culture Collection
BEC	bronchial epithelial cell
BMP	bone morphogenetic protein
BRD	bromodomain BrdU
C4S	chondroitin 4-sulfate
C6S	chondroitin 6-sulfate
CBP	cyclic AMP response element binding protein (CREB) binding protein
CF	cystic fibrosis
CFTR	cystic fibrosis transmembrane conductance regulator
CHST	carbohydrate sulfotransferase
CHST11	carbodhyrate sulfotransferase 11
CHST15	carbohydrate sulfotransferase 15
COPD	chronic obstructive pulmonary disease
CREB	cyclin AMP response element binding protein
CSPG4	chondroitin sulfate proteoglycan 4
DKK	dickkopf Wnt signaling pathway inhibitor
DNMT	DNA methyltransferase
DS	dermatan sulfate
ECAR	extracellular acidification rate
EGF	epidermal growth factor
EGFR	epidermal growth factor receptor
ELISA	enzyme-linked immunosorbent assay
EMT	epithelial-mesenchymal transition
ERK	extracellular signal-regulated kinase
ERT	enzyme replacement therapy
FCCP	carbonyl cyanide 4-(trifluoromethoxy)phenylhydrazone
FFPE	Formalin-fixed paraffin-embedded
FGE	formylglycine generating enzyme
FGly	formylglycine
FPKM	fragments per kilobase of transcript per million mapped reads
GAG	glycosaminoglycan
GALNS	chondroitin-6-sulfatase
GPNMB	transmembrane glycoprotein NMB
GSH	glutathione
G-S-S-G	glutathione disulfide
GWAS	genome-wide association study
HAT	histone acetyltransferase
HDAC	histone deacetylase
HMWK	high molecular weight kininogen
IF	immunofluorescence
IL	interleukin
JNK	c-Jun N-terminal kinase
KD	knockdown
KO	knockout
LGALS3	galectin-3
MAPK	mitogen-activated protein kinase
MCF	Michigan Cancer Foundation
MITF	microphthalmia-associated transcription factor
MLS	Maroteaux-Lamy-Syndrome
MMP	matrix metalloproteinase
MPS VI	Mucopolysaccharidosis VI
MSD	Multiple Sulfatase Deficiency
MUS	methylumbelliferyl sulfate
NCS	nitrocatechol sulfate
MW	molecular weight
OCR	oxygen consumption rate
OE	overexpression
PBS	phosphate-buffered saline
PD-1	programmed death 1
PD-L1	programed death ligand 1
PG	proteoglycan
PHPS1	PTP Inhibitor V, specific for Shp2
PTPN1	tyrosine-protein phosphatase non-receptor type 11; protein-tyrosine phosphatase 1D; Src homology region 2 domain-containing phosphatase 2 (SHP-2); protein-tyrosine phosphatase 2C (PTP-2C)
PD-L1	programmed death ligand 1
QTL	quantitative trait locus
rhARSB	recombinant human arylsulfatase B
RHTIC	Research Histology and Tissue Imaging Core
RRC	Research Resources Center
RTK	receptor tyrosine kinase
sGAG	sulfated glycosaminoglycan
SHP2	PTPN11 (tyrosine-protein phosphatase non-receptor type 11)
siRNA	small interfering RNA
SNP	single nucleotide polymorphism
SOD	superoxide dismutase
Sp1	specificity protein 1
ST	sulfotransferase
SUMF	sulfatase-modifying factor
TCF/LEF	T cell factor/lymphoid enhanced factor transcription factor
TGF	transforming growth factor
TMA	tissue microarray
TNF	tumor necrosis factor

## References

[1] DeSousa J.F., Nader H.B., Dietrich C.P. (1990). Sequential degradation of chondroitin sulfate in molluscs. J. Biol. Chem..

[2] Glaser J.H., Conrad H.E. (1979). Chondroitin SO_4_ catabolism in chick embryo chondrocytes. J. Biol. Chem..

[3] Ingmar B., Wasteson A. (1979). Sequential degradation of a chondroitin sulfate disaccharide by lysosomal enzymes from embryonic-chick epiphysial cartilage. Biochem. J..

[4] Gorham S.D., Cantz M. (1978). Arylsulphatase B, an exo-sulphatase for chondroitin 4-sulphate tetrasaccharide. Hoppe Seylers Z Physiol. Chem..

[5] Cogburn J.N., Silbert J.E. (1986). The effect of penultimate N-acetylgalactosamine 4-sulfate on chondroitin chain elongation. Carbohyd. Res..

[6] Stumpf D.A., Austin J.H., Crocker A.C., LaFrance M. (1973). Mucopolysaccharidosis type VI (Maroteaux-Lamy syndrome). I. Sulfatase B deficiency in tissues. Am. J. Dis. Child..

[7] Fluharty A.L., Stevens R.L., Sanders D.L., Kihara H. (1974). Arylsulfatase B deficiency in Maroteaux-Lamy syndrome cultured fibroblasts. Biochem. Biophys. Res. Commun..

[8] Matalon R., Arbogast B., Dorfman A. (1974). Deficiency of chondroitin sulfate N-acetylgalactosamine 4-sulfate sulfatase in Maroteaux-Lamy syndrome. Biochem. Biophys. Res. Commun..

[9] O’Brien J.F., Cantz M., Spranger J. (1974). Maroteaux-Lamy disease (mucopolysaccharidosis VI), subtype A: Deficiency of a N-acetylgalactosamine-4-sulfatase. Biochem. Biophys. Res. Commun..

[10] D’Avanzo F., Zanetti A., De Filippis C., Tomanin R. (2021). Mucopolysaccharidosis Type VI, an updated overview of the disease. Int. J. Mol. Sci..

[11] Maroteaux P., Leveque B., Marie J., Lamy M. (1963). Une nouvelle dysostose avec elimination urinaire de chondroitine sulfate B. Presse Med..

[12] UniProt P15848 • ARSB_HUMAN. https://www.uniprot.org/uniprotkb/P15848/entry.

[13] Theocharis A.D., Tsara M.E., Papageorgacopoulou N., Karavias D.D., Theocharis D.A. (2000). Pancreatic carcinoma is characterized by elevated content of hyaluronan and chondroitin sulfate with altered disaccharide composition. Biochim. Biophys. Acta.

[14] Bhattacharyya S., Feferman L., Tobacman J.K. (2016). Inhibition of phosphatase activity follows decline in sulfatase activity and leads to transcriptional effects through sustained phosphorylation of transcription factor MITF. PLoS ONE.

[15] Bond C.S., Clements P.R., Ashby S.J., Collyer C.A., Harrop S.J., Hopwood J.J., Guss J.M. (1997). Structure of a human lysosomal sulfatase. Structure.

[16] Ashby S.J., Clements P.R., Guss J.M., Harvey I., Hopwood J.J. (1995). Crystallization and preliminary characterization of human recombinant N-acetylgalactosamine-4-sulfatase. Acta Crystallogr. D Biol. Crystallogr..

[17] Gibson G.J., Saccone G.T., Brooks D.A., Clements P.R., Hopwood J.J. (1987). Human N-acetylgalactosamine-4-sulphate sulphatase. Purification, monoclonal antibody production and native and subunit Mr values. Biochem. J..

[18] Brooks D.A., Robertson D.A., Bindloss C., Litjens T., Anson D.S., Peters C., Morris C.P., Hopwood J.J. (1995). Two site-directed mutations abrogate enzyme activity but have different effects on the conformation and cellular content of the N-acetylgalactosamine 4-sulphatase protein. Biochem. J..

[19] GeneCards ARSB. https://www.genecards.org/cgi-bin/carddisp.pl?gene=ARSB&keywords=arylsulfatase,b.

[20] Modaressi S., Rupp K., von Figura K., Peters C. (1993). Structure of the human arylsulfatase B gene. Biol. Chem. Hoppe Seyler..

[21] Dierks T., Lecca M.R., Schmidt B., von Figura K. (1998). Conversion of cysteine to formylglycine in eukaryotic sulfatases occurs by a common mechanism in the endoplasmic reticulum. FEBS Lett..

[22] Roeser D., Preusser-Kunze A., Schmidt B., Gasow K., Wittmann J.G., Dierks T., von Figura K., Rudolph M.G. (2006). A general binding mechanism for all human sulfatases by the formylglycine-generating enzyme. Proc. Natl. Acad. Sci. USA.

[23] Landgrebe J., Dierks T., Schmidt B., von Figura K. (2003). The human SUMF1 gene, required for posttranslational sulfatase modification, defines a new gene family which is conserved from pro- to eukaryotes. Gene.

[24] Zito E., Fraldi A., Pepe S., Annunziata I., Kobinger G., Di Natale P., Ballabio A., Cosma M.P. (2005). Sulphatase activities are regulated by the interaction of sulphatase-modifying factor 1 with SUMF2. EMBO Rep..

[25] Roeser D., Schmidt B., Preusser-Kunze A., Rudolph M.G. (2007). Probing the oxygen-binding site of the human formylglycine-generating enzyme using halide ions. Acta Crystallogr. D Biol. Crystallogr..

[26] Dierks T., Lecca M.R., Schlotterhose P., Schmidt B., von Figura K. (1999). Sequence determinants directing conversion of cysteine to formylglycine in eukaryotic sulfatases. EMBO J..

[27] Bhattacharyya S., Feferman L., Tobacman J.K. (2014). Arylsulfatase B regulates versican expression by galectin-3 and AP-1 mediated transcriptional effects. Oncogene.

[28] Bhattacharyya S., Feferman L., Han X., Ouyang Y., Zhang F., Linhardt R.J., Tobacman J.K. (2018). Decline in arylsulfatase B expression increases EGFR expression by inhibiting the protein-tyrosine phosphatase SHP2 and activating JNK in prostate cells. J. Biol. Chem..

[29] Bhattacharyya S., Solakyildirim K., Zhang Z., Chen M.L., Linhardt R.J., Tobacman J.K. (2010). Cell-bound IL-8 increases in bronchial epithelial cells after arylsulfatase B silencing due to sequestration with chondroitin-4-sulfate. Am. J. Respir. Cell Mol. Biol..

[30] Bhattacharyya S., Feferman L., Tobacman J.K. (2015). Regulation of chondroitin-4-sulfotransferase (CHST11) expression by opposing effects of arylsulfatase B on BMP4 and Wnt9A. Biochim. Biophys. Acta.

[31] Bhattacharyya S., Kotlo K., Mahdendrabhi T., Danziger R.S., Tobacman J.K. (2010). Arylsulfatase B regulates interaction of chondroitin-4-sulfate and kininogen in renal epithelial cells. BBA: Mol. Basis Dis..

[32] Bhattacharyya S., Feferman L., Tobacman J.K. (2014). Increased expression of colonic Wnt9A through Sp1-mediated transcriptional effects involving arylsulfatase B, chondroitin 4-sulfate, and galectin-3. J. Biol. Chem..

[33] Bhattacharyya S., Feferman L., Terai K., Dudek A.Z., Tobacman J.K. (2017). Decline in arylsulfatase B leads to increased invasiveness of melanoma cells. Oncotarget.

[34] Bhattacharyya S., Tobacman J.K. (2009). Arylsulfatase B regulates colonic epithelial cell migration by effects on MMP9 expression and RhoA activation. Clin. Exp. Metastasis.

[35] Bhattacharyya S., Tobacman J.K. (2014). Hypoxia reduces arylsulfatase B activity and silencing arylsulfatase B replicates and mediates the effects of hypoxia. PLoS ONE.

[36] Bhattacharyya S., Feferman L., Tobacman J.K. (2017). Chondroitin sulfatases differentially regulate Wnt signaling in prostate stem cells through effects on SHP2, phospho-ERK1/2, and Dickkopf Wnt signaling pathway inhibitor (DKK3). Oncotarget.

[37] Bhattacharyya S., Feferman L., Tobacman J.K. (2019). Dihydrotestosterone inhibits arylsulfatase B and Dickkopf Wnt signaling pathway inhibitor (DKK)-3 leading to enhanced Wnt signaling in prostate epithelium in response to stromal Wnt3A. Prostate.

[38] Bhattacharyya S., Feferman L., Han X., Xia K., Zhang F., Linhardt R.J., Tobacman J.K. (2020). Increased CHST15 follows decline in arylsulfatase B (ARSB) and disinhibition of non-canonical WNT signaling: Potential impact on epithelial and mesenchymal identity. Oncotarget.

[39] Bhattacharyya S., Tobacman J.K. (2007). Increased arylsulfatase B activity in cystic fibrosis cells following correction of CFTR. Clin. Chim. Acta.

[40] Mitsunaga-Nakatsubo K., Kusunoki S., Kawakami H., Akasaka K., Akimoto Y. (2009). Cell-surface arylsulfatase A and B on sinusoidal endothelial cells, hepatocytes, and Kupffer cells in mammalian livers. Med. Mol. Morphol..

[41] Prabhu S.V., Bhattacharyya S., Guzman-Hartman G., Macias V., Kajdacsy-Balla A., Tobacman J.K. (2011). Extra-lysosomal localization of arylsulfatase B in human colonic epithelium. J. Histochem. Cytochem..

[42] Feferman L., Bhattacharyya S., Deaton R., Gann P., Guzman G., Kajdacsy-Balla A., Tobacman J.K. (2013). Arylsulfatase B (N-acetylgalactosamine-4-sulfatase): Potential role as a biomarker in prostate cancer. Prostate Cancer Prostatic Dis..

[43] Feferman L., Deaton R., Bhattacharyya S., Xie H., Gann P.H., Melamed J., Tobacman J.K. (2017). Arylsulfatase B is reduced in prostate cancer recurrences. Cancer Biomark..

[44] Bhattacharyya S., Solakyildirim K., Zhang Z., Linhardt R.J., Tobacman J.K. (2009). Chloroquine reduces arylsulphatase B activity and increases chondroitin-4-sulphate: Implications for mechanisms of action and resistance. Malar. J..

[45] Murata F., Nagata T., Spicer S.S. (1975). Fine structural localization of arylsulfatase B activity in the rabbit blood platelets. Histochemistry.

[46] Makita T., Sandborn E.B. (1971). Ultrastructural localization of arylsulfatase B in mitochondria of epithelial cells of the proximal convoluted tubules of the rat kidney. Experientia.

[47] Luo M., Chen J., Li S., Sun H., Zhang Z., Fu Q., Li J., Wang J., Hughes C.E., Caterson B. (2014). Changes in the metabolism of chondroitin sulfate glycosaminoglycans in articular cartilage from patients with Kashin-Beck disease. Osteoarthr. Cartil..

[48] Stevens R.L., Fluharty A.L., Killgrove A.R., Kihara Y. (1977). Arylsulfatase of human tissue. Studies on a form of arylsulfatase B found predominantly in brain. Biochim. Biophys. Acta.

[49] Kung M.P., Roth J.A. (1987). Cellular localization of soluble and membrane-bound forms of arylsulfatase in rat brain. Brain Res..

[50] Lakshmi S., Balasubramanian A.S. (1980). Soluble arylsulfatases of human brain and some characteristics of the brain-specific arylsulfatase B_m_. Biochim. Biophys. Acta..

[51] The Human Protein Atlas. https://www.proteinatlas.org/ENSG00000113273-ARSB/tissue.

[52] Bhattacharyya S., Feferman L., Tobacman J.K. (2016). Restriction of aerobic metabolism by acquired or innate Arylsulfatase B deficiency: A new approach to the Warburg effect. Sci. Rep..

[53] Dzialoszynski L.M. (1955). 4-Nitrocatechol sulfate as a substrate for the assay of aryl sulfatase. Acta Biochim. Pol..

[54] Roy A.B. (1987). Arylsulfatases, colorimetric and fluorimetric assays. Methods Enzymol..

[55] Pungor E., Hague C.M., Chen G., Lemontt J.F., Dvorak-Ewell M., Prince W.S. (2009). Development of a functional bioassay for arylsulfatase B using the natural substrates of the enzyme. Anal. Biochem..

[56] Kumar A.B., Spacil Z., Ghomashchi F., Masi S., Sumida T., Ito M., Turecek F., Scott C.R., Gelb M.H. (2015). Fluorimetric assays for N-acetylgalactosamine-6-sulfatase and arylsulfatase B based on the natural substrates for confirmation of mucopolysaccharidoses types IVA and VI. Clin. Chim. Acta.

[57] Hein L.K., Meikle P.J., Dean C.J., Bockmann M.R., Auclair D., Hopwood J.J., Brooks D.A. (2005). Development of an assay for the detection of mucopolysaccharidosis type VI patients using dried blood-spots. Clin. Chim. Acta.

[58] Ferrero G.B., Pagliardini S., Veljkovic A., Porta F., Bena C., Tardivo I., Restagno G., Silengo M.C., Bignamini E. (2008). In vivo specific reduction of arylsulfatase B enzymatic activity in children with cystic fibrosis. Mol. Genet. Metab..

[59] Fluharty A.L., Stevens R.L., Goldstein E.B., Kihara H. (1979). The activity of arylsulfatase A and B on tyrosine O-sulfates. Biochim. Biophys. Acta.

[60] Bakhru-Kishore R., Kelly S. (1977). A microfluorometric assay of the lysosomal arylsulfatases in leukocytes. Clin. Chim Acta.

[61] Mercelis R., Van Elsen A.F., Leroy J.G. (1979). Arylsulfatases A and B in human diploid fibroblasts: Differential assay with 4-methylumbelliferylsulfate and AgNO_3_. Clin. Chim. Acta.

[62] Chang P.L., Rosa N.E., Davidson R.G. (1981). Differential assay of arylsulfatases A and B activities: A sensitive method for cultured human cells. Anal. Biochem..

[63] Rao G.J., Christe M.E. (1984). Inhibition of rabbit liver arylsulfatase B by phosphate esters. Biochim. Biophys. Acta.

[64] Wòjczyk B. (1986). Lysosomal arylsulfatases A and B from horse blood leukocytes: Purification and physico-chemical properties. Biol. Cell.

[65] Baum H., Dodgson K.S., Spencer B. (1959). The assay of arylsulphatases A and B in human urine. Clin. Chim. Acta.

[66] Baum H., Dodgson K.S. (1958). Arylsulphate synthesis and the arylsulphatases. Nature.

[67] Gold E.W., Gussler D., Schwartz E.R. (1976). Enzymes from human articular cartilage: Isolation of arylsulfatase B and its comparison with arylsulfatase A. Connect Tissue Res..

[68] Wasserman S.I., Austen K.F. (1976). Arylsulfatase B of human lung. Isolation, characterization, and interaction with slow-reacting substance of anaphylaxis. J. Clin. Investig..

[69] Schwartz E.R., Adamy L. (1976). Effect of ascorbic acid on arylsulfatase A and B activities in human chondrocyte cultures. Connect Tissue Res..

[70] Kotlo K., Bhattacharyya S., Yang B., Feferman L., Tejaskumar S., Linhardt R., Danziger R., Tobacman J.K. (2013). Impact of salt exposure on N-acetylgalactosamine-4-sulfatase (arylsulfatase B) activity, glycosaminoglycans, kininogen, and bradykinin. Glycoconj. J..

[71] Feferman L., Bhattacharyya S., Birch L., Prins G.S., Tobacman J.K. (2014). Differential effects of estrogen exposure on arylsulfatase B, galactose-6-sulfatase, and steroid sulfatase in rat prostate development. J. Steroid Biochem. Mol. Biol..

[72] Bhattacharyya S., Tobacman J.K. (2007). Steroid sulfatase, arylsulfatases A and B, galactose-6-sulfatase, and iduronate sulfatase in mammary cells and effects of sulfated and non-sulfated estrogens on sulfatase activity. J. Steroid Biochem. Mol. Biol..

[73] Bhattacharyya S., Tobacman J.K. (2012). Molecular signature of kappa-carrageenan mimics chondroitin-4-sulfate and dermatan sulfate and enables interaction with arylsulfatase B. J. Nutr. Biochem..

[74] Yang B., Bhattacharyya S., Linhardt R., Tobacman J. (2012). Exposure to common food additive carrageenan leads to reduced sulfatase activity and increase in sulfated glycosaminoglycans in human epithelial cells. Biochimie.

[75] Zhang X., Bhattacharyya S., Kusumo H., Goodlett C.R., Tobacman J.K., Guizzetti M. (2014). Arylsulfatase B modulates neurite outgrowth via astrocyte chondroitin-4-sulfate: Dysregulation by ethanol. Glia.

[76] Achur R.N., Valiyaveettil M., Gowda D.C. (2003). The low sulfated chondroitin sulfate proteoglycans of human placenta have sulfate group-clustered domains that can efficiently bind Plasmodium falciparum-infected erythrocytes. J. Biol. Chem..

[77] Muthusamy A., Achur R.N., Bhavanandan V.P., Fouda G.G., Taylor D.W., Gowda D.C. (2004). Plasmodium falciparum-infected erythrocytes adhere both in the intervillous space and on the villous surface of human placenta by binding to the low sulfated chondroitin sulfate proteoglycan receptor. Am. J. Pathol..

[78] Spliid C.B., Toledo A.G., Sanderson P., Mao Y., Gatto F., Gustavsson T., Choudhary S., Saldanha A.L., Vogelsang R.P., Gögenur I. (2021). The specificity of the malarial VAR2CSA protein for chondroitin sulfate depends on 4-O-sulfation and ligand accessibility. J. Biol. Chem..

[79] Wang K., Dagil R., Lavstsen T., Misra S.K., Spliid C.B., Wang Y., Gustavsson T., Sandoval D.R., Vidal-Calvo E.E., Choudhary S. (2021). Cryo-EM reveals the architecture of placental malaria VAR2CSA and provides molecular insight into chondroitin sulfate binding. Nat. Commun..

[80] Bhattacharyya S., Feferman L., Tobacman J.K. (2019). Distinct effects of carrageenan and high-fat consumption on the mechanisms of insulin resistance in nonobese and obese models of Type 2 diabetes. J. Diabetes Res..

[81] Li P., Liu S., Lu M., Bandyopadhyay G., Oh D., Imamura T., Johnson A.M.F., Sears D., Shen Z., Cui B. (2016). Hematopoietic-derived galectin-3 causes cellular and systemic insulin resistance. Cell.

[82] Feferman L., Bhattacharyya S., Oates E., Haggerty N., Wang T., Varady K., Tobacman J.K. (2020). Carrageenan-free diet shows improved glucose tolerance and insulin signaling in prediabetes: A randomized, pilot clinical trial. J. Diabetes Res..

[83] Bhattacharyya S., Feferman L., Sharma G., Tobacman J.K. (2018). Increased GPNMB, phosphoRK1/2, and MMP-9 in cystic fibrosis in association with reduced arylsulfatase B. Mol. Genet. Metab..

[84] Iwaki J., Minamisawa T., Tateno H., Kominami J., Suzuki K., Nishi N., Nakamura T., Hirabayashi J. (2008). Desulfated galactosaminoglycans are potential ligands for galectins: Evidence from frontal affinity chromatography. Biochem. Biophys. Res. Commun..

[85] Tobacman J.K., Bhattacharyya S., Feferman L. (2020). Decline in Arylsulfatase B (ARSB) increases PD-L1 expression in melanoma, hepatic, prostate, and mononuclear cells. Cancer Res..

[86] Hellmuth K., Grosskopf S., Lum C.T., Würtele M., Röder N., von Kires J.P., Rosario M., Rademann J., Birchmeier W. (2008). Specific inhibitors of the protein tyrosine phosphatase Shp2 Identified by high-throughput docking. Proc. Natl. Acad. Sci. USA.

[87] Tobacman J.K., Bhattacharyya S., Feferman L. (2021). Chondroitin sulfatases and transcription factors Gli, Tcf/Lef, and c-Myc in prostate stem cells [abstract]. Cancer Res..

[88] Sharma G., Burke J., Bhattacharyya S., Sharma N., Katyal S., Park R.L., Tobacman J. (2013). Reduced arylsulfatase B activity in leukocytes from cystic fibrosis patients. Pediatr. Pulmonol..

[89] Bhattacharyya S., Feferman L., Tobacman J.K. (2016). Effect of CFTR modifiers on arylsulfatase B activity in cystic fibrosis and normal human bronchial epithelial cells. Pulm. Pharmacol. Ther..

[90] Taya M., Hammes S.R. (2018). Glycoprotein non-metastatic melanoma protein B (GPNMB) and cancer: A novel potential therapeutic target. Steroids.

[91] Rose A.A.N., Biondini M., Curiel R., Siegel P.M. (2017). Targeting GPNMB with glembatumumab vedotin: Current developments and future opportunities for the treatment of cancer. Pharmacol. Ther..

[92] An C.H., Kim S.S., Kang M.R., Kim Y.R., Kim H.S., Yoo N.J., Lee S.H. (2010). Frameshift mutations of ATBF1, WNT9A, CYLD, and PARK2 in gastric and colorectal carcinomas with high microsatellite instability. Pathology.

[93] Kirikoshi H., Sekihara H., Katoh M. (2001). Expression of WNT14 and WNT14B mRNAs in human cancer, up-regulation of WNT14 by IFN γ and up-regulation of WNT14B by β-estradiol. Int. J. Oncol..

[94] Bhattacharyya S., Kotlo K., Shukla S., Danziger R.S., Tobacman J.K. (2008). Distinct effects of N-acetylgalactosamine-4-sulfatase and galactose-6-sulfatase expression on chondroitin sulfates. J. Biol. Chem..

[95] Tessitore A., Pirozzi M., Auricchio A. (2009). Abnormal autophagy, ubiquitination, inflammation and apoptosis are dependent upon lysosomal storage and are useful biomarkers of mucopolysaccharidosis VI. Pathogenetics.

[96] Gasper P.W., Thrall M.A., Wenger D.A., Macy D.W., Ham L., Dornsife R.E., McBiles K., Quackenbush S.L., Kesel M.L., Gillette E.L. (1984). Correction of feline arylsulphatase B deficiency (mucopolysaccharidosis VI) by bone marrow transplantation. Nature.

[97] Tobacman J.K. (2003). Does deficiency of arylsulfatase B have a role in cystic fibrosis?. Chest.

[98] Kovacs Z., Jung I., Szalman K., Banias L., Bara T., Gurzu S. (2019). Interaction of arylsulfatases A and B with maspin: A possible explanation for dysregulation of tumor cell metabolism and invasive potential of colorectal cancer. World J. Clin. Cases.

[99] Ricciardelli C., Mayne K., Sykes P.J., Raymond W.A., McCaul K., Marshall V.R., Tilley W.D., Skinner J.M., Horsfall D.J. (1997). Elevated stromal chondroitin sulfate glycosaminoglycan predicts progression in early-stage prostate cancer. Clin. Cancer Res..

[100] Ricciardelli C., Mayne K., Sykes P.J., Raymond W.A., McCaul K., Marshall V.R., Horsfall D.J. (1998). Elevated levels of versican but not decorin predict disease progression in early-stage prostate cancer. Clin. Cancer Res..

[101] Ricciardelli C., Sakko A.J., Ween M.P., Russell D.L., Horsfall D.J. (2009). The biological role and regulation of versican levels in cancer. Cancer Metastasis Rev..

[102] Figlioli G., Köhler A., Chen B., Elisei R., Romei C., Cipollini M., Cristaudo A., Bambi F., Paolicchi E., Hoffmann P. (2014). Novel genome-wide association study-based candidate loci for differentiated thyroid cancer risk. J. Clin. Endocrinol. Metab..

[103] Wolańska M., Sobolewski K., Cechowska-Pasko M., Jaworski S. (2003). The activities of some glycosaminoglycan-degrading enzymes in uterine leiomyomas. Eur. J. Obstet. Gynecol. Reprod. Biol..

[104] Yoo M., Khaled M., Gibbs K.M., Kim J., Kowalewski B., Dierks T., Schachner M. (2013). Arylsulfatase B improves locomotor function after mouse spinal cord injury. PLoS ONE.

[105] Park H.H., Kim Y.M., Anh Hong L.T., Kim H.S., Kim S.H., Jin X., Hwang D.H., Kwon M.J., Song S.C., Kim B.G. (2022). Dual-functional hydrogel system for spinal cord regeneration with sustained release of arylsulfatase B alleviates fibrotic microenvironment and promotes axonal regeneration. Biomaterials.

[106] Pearson C.S., Mencio C.P., Barber A.C., Martin K.R., Geller H.M. (2018). Identification of a critical sulfation in chondroitin that inhibits axonal regeneration. Elife.

[107] Wang H., Katagiri Y., McCann T.E., Unsworth E., Goldsmith P., Yu Z.X., Tan F., Santiago L., Mills E.M., Wang Y. (2008). Chondroitin-4-sulfation negatively regulates axonal guidance and growth. J. Cell Sci..

[108] Pearson C.S., Solano A.G., Tilve S.M., Mencio C.P., Martin K.R., Geller H.M. (2020). Spatiotemporal distribution of chondroitin sulfate proteoglycans after optic nerve injury in rodents. Exp. Eye Res..

[109] Zhang J., Liang H., Zhu L., Gan W., Tang C., Li J., Xu R. (2018). Expression and distribution of Arylsulfatase B are closely associated with neuron death in SOD1 G93A transgenic mice. Mol. Neurobiol..

[110] Bhattacharyya S., Zhang X., Feferman L., Johnson D., Tortella F.C., Guizzetti M., Tobacman J.K. (2015). Decline in arylsulfatase B and Increase in chondroitin 4-sulfotransferase combine to increase chondroitin 4-sulfate in traumatic brain injury. J. Neurochem..

[111] Potkin S.G., Guffanti G., Lakatos A., Turner J.A., Kruggel F., Fallon J.H., Saykin A.J., Orro A., Lupoli S., Salvi E. (2009). Hippocampal atrophy as a quantitative trait in a genome-wide association study identifying novel susceptibility genes for Alzheimer’s disease. PLoS ONE.

[112] Li Y., Xu C., Schubert D. (1999). The up-regulation of endosomal-lysosomal components in amyloid beta-resistant cells. J. Neurochem..

[113] Blake M.R., Parrish D.C., Staffenson M.A., Sueda S., Woodward W.R., Habecker B.A. (2022). Chondroitin sulfate proteoglycan 4,6 sulfation regulates sympathetic nerve regeneration after myocardial infarction. eLife.

[114] Tzankov A., Bhattacharyya S., Kotlo K., Tobacman J.K. (2022). Increase in chondroitin sulfate and decline in Arylsulfatase B may contribute to pathophysiology of COVID-19 respiratory failure. Pathobiology.

[115] Weller P.F., Ottesen E.A., Goetzl E.J. (1981). Sequential alterations in the human eosinophil content of arylsulfatase B during therapy of Bacroftian filariasis. Clin. Immunol. Immunopathol..

[116] Balbaa M., El-Kersh M., Mansour H., Yacout G., Ismail M., Malky A., Bassiouny K., Abdel-Monem N., Kandeel K. (2004). Activity of some hepatic enzymes in schistosomiasis and concomitant alteration of arylsulfatase B. J. Biochem. Mol. Biol..

[117] Pohl S., Angermann A., Jeschke A., Hendrickx G., Yorgan T.A., Makrypidi-Fraune G., Steigert A., Kuehn S.C., Rolvien T., Schweizer M. (2018). The lysosomal protein Arylsulfatase B Is a key enzyme involved in skeletal turnover. J. Bone Miner. Res..

[118] Simonaro C.M., D’Angelo M., He X., Eliyahu E., Shtraizent N., Haskins M.E., Schuchman E.H. (2008). Mechanism of glycosaminoglycan-mediated bone and joint disease: Implications for the mucopolysaccharidoses and other connective tissue diseases. Am. J. Pathol..

[119] Simonaro C.M., Haskins M.E., Schuchman E.H. (2001). Articular chondrocytes from animals with a dermatan sulfate storage disease undergo a high rate of apoptosis and release nitric oxide and inflammatory cytokines: A possible mechanism underlying degenerative joint disease in the mucopolysaccharidoses. Lab. Investig..

[120] Colofiore J.R., Schwartz E.R. (1986). Monensin stimulation of arylsulfatase B activity in human chondrocytes. J. Orthop. Res..

[121] Golda A., Jurecka A., Gajda K., Tylki-Szymańska A., Lalik A. (2015). Human pulmonary artery endothelial cells in the model of mucopolysaccharidosis VI present a prohypertensive phenotype used for studies of pulmonary hypertension. Mol. Genet. Metab. Rep..

[122] Seo M., Qiu W., Bailey W., Criner G.J., Dransfield M.T., Fuhlbrigge A.L., Reilly J.J., Scholand M.B., Castaldi P., Chase R. (2018). Genomics and response to long-term oxygen therapy in chronic obstructive pulmonary disease. J. Mol. Med..

[123] Strauch O.F., Stypmann J., Reinheckel T., Martinez E., Haverkamp W., Peters C. (2003). Cardiac and ocular pathologies in a mouse model of mucopolysaccharidosis type VI. Pediatr Res..

[124] Sleeper M.M., Kusiak C.M., Shofer F.S., O’Donnell P., Bryan C., Ponder K.P., Haskins M.E. (2008). Clinical characterization of cardiovascular abnormalities associated with feline mucopolysaccharidosis I and VI. J. Inherit. Metab. Dis..

[125] Zhao R.R., Ackers-Johnson M., Stenzig J., Chen C., Ding T., Zhou Y., Wang P., Ng S.L., Li P.Y., Teo G. (2018). Targeting chondroitin sulfate glycosaminoglycans to treat cardiac fibrosis in pathological remodeling. Circulation.

[126] Wang Q., Yu X., Dou L., Huang X., Zhu K., Guo J., Yan M., Wang S., Man Y., Tang W. (2019). miR-154-5p Functions as an important regulator of Angiotensin II-mediated heart remodeling. Oxid. Med. Cell. Longev..

[127] Watanabe K., Arumugam S., Sreedhar R., Thandavarayan R.A., Nakamura T., Nakamura M., Harima M., Yoneyama H., Suzuki K. (2015). Small interfering RNA therapy against carbohydrate sulfotransferase 15 inhibits cardiac remodeling in rats with dilated cardiomyopathy. Cell Signal..

[128] Kowalewski R., Sobolewski K., Malkowski A., Gacko M., Rutkowska I. (2008). Glycosaminoglycan-degrading enzymes in the varicose vein wall. Int. Angiol..

[129] Biros E., Moran C.S., Maguire J., Holliday E., Levi C., Golledge J. (2017). Upregulation of arylsulfatase B in carotid atherosclerosis is associated with symptoms of cerebral embolization. Sci. Rep..

[130] Dodgson K.S., Wynn C.H. (1958). Studies on sulphatases. 19. The purification and properties of arylsulphatase B of human liver. Biochem. J..

[131] McKusick V. (1969). The nosology of the mucopolysaccharidoses. Am. J. Med..

[132] Dzialoszyński L.M., Kroll J.L., Fröhlich A. (1966). Arylsulphatase activity of some malignant tumors. Clin. Chim. Acta.

[133] Baron R.W., Neufeld E.F. (1972). A distinct biochemical deficit in the Maroteaux-Lamy syndrome (mucopolysaccharidosis VI). J. Pediatr..

[134] Gniot-Szulzycka J. (1972). Human placenta arylsulphatase B purification and separation into subfractions. Acta Biochim. Pol..

[135] Németh-Csóka M. (1969). The sulphokinase, arylsulphatase-B and acid phosphatase activities of carrageenin granuloma in rats of different ages. Gerontologia.

[136] Shapira E., DeGregorio R.R., Matalon R., Nadler H.L. (1975). Reduced arylsulfatase B activity of the mutant enzyme protein in Maroteaux-Lamy syndrome. Biochem. Biophys. Res. Commun..

[137] Beratis N.G., Turner B.M., Weiss R., Hirschhorn K. (1975). Arylsulfatase B deficiency in Maroteaux-Lamy syndrome: Cellular studies and carrier identification. Pediatr. Res..

[138] NORD Rare Disease Database. https://rarediseases.org/rare-diseases/maroteaux-lamy-syndrome/.

[139] Giugliani R., Lampe C., Guffon N., Ketteridge D., Leão-Teles E., Wraith J.E., Jones S.A., Piscia-Nichols C., Lin P., Quartel A. (2014). Natural history and galsulfase treatment in mucopolysaccharidosis VI (MPS VI, Maroteaux-Lamy syndrome)—10-year follow-up of patients who previously participated in an MPS VI Survey Study. Am. J. Med. Genet. A.

[140] Krivit W., Pierpont M.E., Ayaz K., Tsai M., Ramsay N.K., Kersey J.H., Weisdorf S., Sibley R., Snover D., McGovern M.M. (1984). Bone-marrow transplantation in the Maroteaux-Lamy syndrome (mucopolysaccharidosis type VI). Biochemical and clinical status 24 months after transplantation. N. Engl. J. Med..

[141] Lee V., Li C.K., Shing M.M., Chik K.W., Lam C.W., Tsang K.S., Pong H., Huen K.F., Yuen P.M. (2000). Umbilical cord blood transplantation for Maroteaux-Lamy syndrome (mucopolysaccharidosis type VI). Bone Marrow Transplant..

[142] Harmatz P., Giugliani R., Schwartz I., Guffon N., Teles E.L., Miranda M.C., Wraith J.E., Beck M., Arash L., Scarpa M. (2006). Enzyme replacement therapy for mucopolysaccharidosis VI: A phase 3, randomized, double-blind, placebo-controlled, multinational study of recombinant human N-acetylgalactosamine 4-sulfatase (recombinant human arylsulfatase B or rhARSB) and follow-on, open-label extension study. J. Pediatr..

[143] Peters C., Rommerskirch W., Modaressi S., von Figura K. (1991). Restoration of arylsulphatase B activity in human mucopolysaccharidosis-type-VI fibroblasts by retroviral-vector-mediated gene transfer. Biochem. J..

[144] Ferla R., Alliegro M., Dell’Anno M., Nusco E., Cullen J.M., Smith S.N., Wolfsberg T.G., O’Donnell P., Wang P., Nguyen A.D. (2020). Low incidence of hepatocellular carcinoma in mice and cats treated with systemic adeno-associated viral vectors. Mol. Ther. Methods Clin. Dev..

[145] Crawley A.C., Brooks D.A., Muller V.J., Petersen B.A., Isaac E.L., Bielicki J., King B.M., Boulter C.D., Moore A.J., Fazzalari N.L. (1996). Enzyme replacement therapy in a feline model of Maroteaux-Lamy syndrome. J. Clin. Investig..

[146] Jezyk P.F., Haskins M.E., Patterson D.F., Mellman W.J., Greenstein M. (1977). Mucopolysaccharidosis in a cat with arylsulfatase B deficiency: A model of Maroteaux-Lamy syndrome. Science.

[147] Haskins M.E., Jezyk P.F., Desnick R.J., Patterson D.F. (1981). Animal model of human disease: Mucopolysaccharidosis VI Maroteaux-Lamy syndrome, Arylsulfatase B-deficient mucopolysaccharidosis in the Siamese cat. Am. J. Pathol..

[148] Cosma M.P., Pepe S., Annunziata I., Newbold R.F., Grompe M., Parenti G., Ballabio A. (2003). The multiple sulfatase deficiency gene encodes an essential and limiting factor for the activity of sulfatases. Cell.

[149] Dierks T., Schmidt B., Borissenko L.V., Peng J., Preusser A., Mariappan M., von Figura K. (2003). Multiple sulfatase deficiency is caused by mutations in the gene encoding the human C(alpha)-formylglycine generating enzyme. Cell.

[150] Fraldi A., Biffi A., Lombardi A., Visigalli I., Pepe S., Settembre C., Nusco E., Auricchio A., Naldini L., Ballabio A. (2007). SUMF1 enhances sulfatase activities in vivo in five sulfatase deficiencies. Biochem. J..

[151] Fraldi A., Zito E., Annunziata F., Lombardi A., Cozzolino M., Monti M., Spampanato C., Ballabio A., Pucci P., Sitia R. (2008). Multistep, sequential control of the trafficking and function of the multiple sulfatase deficiency gene product, SUMF1 by PDI, ERGIC-53 and Erp44. Hum. Mol. Genet..

[152] Schlotawa L., Adang L.A., Radhakrishnan K., Ahrens-Nicklas R.C. (2020). Multiple Sulfatase Deficiency: A disease comprising mucopolysaccharidosis, sphingolipidosis, and more caused by a defect in posttranslational modification. Int. J. Mol. Sci..

[153] MedlinePlus Multiple Sulfatase Deficiency. https://medlineplus.gov/genetics/condition/multiple-sulfatase-deficiency/#.

[154] Mukherji R.N., Moss P.H., Heffernan C.K. (1976). Is cystic fibrosis an acid mucopolysaccharidosis?. Arch. Dis. Child..

[155] Matalon R., Dorfman A. (1969). Acid mucopolysaccharides in cultured human fibroblasts. Lancet.

[156] Mason K.A., Rogol A.D. (2022). Trends in growth and maturation in children with cystic fibrosis throughout nine decades. Front. Endocrinol..

[157] MedlinePlus Cystic Fibrosis. https://medlineplus.gov/ency/article/000107.htm#.

[158] NORD Rare Disease Database. Cystic Fibrosis. https://rarediseases.org/rare-diseases/cystic-fibrosis/.

[159] Bosch L., Bosch B., De Boeck K., Nawrot T., Meyts I., Vanneste D., Le Bourlegat C.A., Croda J., da Silva Filho L.V.R.F. (2017). Cystic fibrosis carriership and tuberculosis: Hints toward an evolutionary selective advantage based on data from the Brazilian territory. BMC Infect. Dis..

[160] Poolman E.M., Galvani A.P. (2007). Evaluating candidate agents of selective pressure for cystic fibrosis. J. R. Soc. Interface.

[161] Maisonneuve P., Marshall B.C., Knapp E.A., Lowenfels A.B. (2013). Cancer risk in cystic fibrosis: A 20-year nationwide study from the United States. J. Natl. Cancer Inst..

[162] Zhang J.T., Jiang X.H., Xie C., Cheng H., Da Dong J., Wang Y., Fok K.L., Zhang X.H., Sun T.T., Tsang L.L. (2013). Downregulation of CFTR promotes epithelial-to-mesenchymal transition and is associated with poor prognosis of breast cancer. Biochim. Biophys. Acta.

[163] Appelt D., Steinkamp G., Ellemunter H. (2022). Cancer in Cystic Fibrosis: Do Not Neglect Gynecologic Cancers. Chest.

[164] Morgan L.R., Samuels M.S., Thomas W., Krementz E.T., Meeker W. (1975). Arylsulfatase B in colorectal cancer. Cancer.

[165] Schatoff E.M., Leach B.I., Dow L.E. (2017). Wnt signaling and colorectal cancer. Curr. Colorectal Cancer Rep..

[166] Scherstén T., Wahlqvist L., Jilderos B. (1971). Lysosomal enzyme activity in liver tissue, kidney tissue, and tumor tissue from patients with renal carcinoma. Cancer.

[167] Morgan I.R., Reehlmann N.J., Maddux R., Rodriguez L., Gidman G., Samuels M.S., Krementz E.T. (1971). Arylsulphatase activity of human lung, liver and kidney neoplasms. Clin. Chim. Acta.

[168] Gasa S., Balbaa M., Nakamura M., Yonemori H., Makita A. (1987). Phosphorylation of human lysosomal arylsulfatase B by cAMP-dependent protein kinase. Different sites of phosphorylation between normal and cancer tissues. J. Biol. Chem..

[169] Kovacs Z., Jung I., Gurzu S. (2019). Arylsulfatases A and B: From normal tissues to malignant tumors. Pathol. Res. Pract..

[170] Igarashi M. (1983). The activity of arylsulphatases in serum of patients with several bullous diseases. Tohoku J. Exp. Med..

[171] Romanowicz L., Bańkowski E., Sobolewski K., Jaworski S. (1999). Activities of some glycosaminoglycan- degrading enzymes in Wharton’s jelly and their alteration in EPH-gestosis (Pre-eclampsia). Biol. Neonate.

[172] Takagi S., Maguti S., Tsuga T., Yoshimura T., Fukuda S. (1989). Activity of serum arylsulfatase B in nasal allergy patients. Nihon Jibiinkoka Gakkai Kaiho.

[173] Kraaijenhagen R.J., Schipper-Kester G.P., Rijksen G., de Gast G.C., Staal G.E. (1982). Lysosomal enzymes in normal and leukemic B lymphocytes. Clin. Chim. Acta.

[174] Uehara Y., Gasa S., Makita A., Sakurada K., Miyazaki T. (1983). Lysosomal arylsulfatases of human leukocytes: Increment of phosphorylated B variants in chronic myelogenous leukemia. Cancer Res..

